# Supplementary material for: Comparative Analysis Based on Transcriptomics and Metabolomics Data Reveal Differences between Emmer and Durum Wheat in Response to Nitrogen Starvation
Source: Int J Mol Sci. 2021 Apr 30;22(9):4790. doi: 10.3390/ijms22094790 (PMC8124848; doi:10.3390/ijms22094790)
Supplement: Supplementary file 1 [file ijms-22-04790-s001.zip › Suppl.Table S1.pdf]

Table S1. Reads and mapping results.

Specie	Genotype	Nitrogen Level	Reads Total Number	Reads Total Number (cleaned)	Reads Mapped	Reads mean	Reads Mapped mean	%
**emmer** *Triticum turgidum* ssp. *dicoccum*	Molise	+N	12,426,327	11,729,149	8,634,292	14,514,858	10,578,245	72.9
			15,048,113	13,952,143	10,324,849			
			14,903,919	14,109,719	9,863,795			
			19,540,053	18,268,422	13,490,043			
		–N	14,549,043	13,877,108	9,621,513	12,123,763	8,512,069	70.2
			15,385,878	14,271,168	10,653,558			
			10,419,996	9,732,724	6,506,270			
			11,085,787	10,614,052	7,266,933			
**durum wheat** *Triticum turgidum* ssp. *turgidum* convar *durum*	Simeto	+N	11,679,027	10,989,390	7,657,401	12,161,459	8,247,526	67.8
			14,556,720	13,748,679	9,989,064			
			13,016,891	12,037,935	8,523,540			
			12,548,405	11,869,831	6,820,097			
		–N	14,047,180	13,176,130	9,423,828	13,197,161	9,291,518	70.4
			16,677,090	15,519,511	10,966,290			
			15,471,231	14,636,407	10,024,095			
			9,905,802	9,456,597	6,751,858			
