# Supplementary material for: Comparative Analysis Based on Transcriptomics and Metabolomics Data Reveal Differences between Emmer and Durum Wheat in Response to Nitrogen Starvation
Source: Int J Mol Sci. 2021 Apr 30;22(9):4790. doi: 10.3390/ijms22094790 (PMC8124848; doi:10.3390/ijms22094790)
Supplement: Supplementary file 1 [file ijms-22-04790-s001.zip › Suppl.Table S2.pdf]

Table S2. Differentially expressed genes in common or specific in emmer (cv. Molise Sel. Colli) and in durum wheat (cv. Simeto) under nitrogen starvation. Key genes involved in nitrogen metabolism, carbon metabolism, transporters, transcription factors, kinases, and stress-related genes are reported. In blue are represented DEGs up regulated and in red DEGs down regulated.

N metabolism and key enzymes	Gene ID	Description	common	Molise Sel. Colli	Simeto
N	Traes_6AS_AB7CDF374	Nitrate reductase			x
	Traes_6AS_148C79718	Nitrate reductase			x
	Traes_5BL_7E69810D5	Asparagine synthetase [glutamine-hydrolyzing]1	x		
	Traes_6AL_0F8A39C3D	Aspartate aminotransferase, chloroplastic	x		
	Traes_3AL_B137CC856	Aspartate aminotransferase 3, chloroplastic			x
	Traes_7BL_0029CE4EA	Aspartate aminotransferase			x
	Traes_6AL_2017727C4	Glutamine synthetase			x
	Traes_6BL_51A18A82E	Glutamine synthetase			x
	Traes_6BL_95C7F7123	Glutamine synthetase			x
	Traes_2AL_3F84846F1	Glutamate dehydrogenase			x
Amino acids metabolism	Traes_2BL_6552196A1	3-isopropylmalate dehydrogenase			x
	Traes_2AL_B4C264AB8	3-isopropylmalate dehydrogenase	x		
	Traes_5AL_0002B669C	Beta-ureidopropionase			x
	Traes_5BL_AA36BC1E0	Beta-ureidopropionase			x
	Traes_2BL_0EF162538	Agmatine deiminase			x
	Traes_2BL_2768AE3B1	Aminomethyltransferase			x
	Traes_2BL_79F69A456	Aminomethyltransferase			x
	Traes_6BS_8C5C8E9E2	Aminopeptidase	x		
	Traes_6AS_ADCD6AB01	Aminopeptidase M1	x		
	Traes_4BL_F15D3655D	Arginine biosynthesis bifunctional protein ArgJ, chloroplastic	x		
	Traes_4AS_641F6B847	Arginine biosynthesis bifunctional protein ArgJ, chloroplastic	x		
	Traes_1BS_DB2EFB90D	Arginine decarboxylase	x		

Traes_1BL_708C02E25	Branched-chain-amino-acid aminotransferase		x	
Traes_2BL_FFF96039E	Branched-chain-amino-acid aminotransferase			x
Traes_5BL_537388393	Carbamoyl-phosphate synthase small chain, chloroplastic		x	
Traes_4AL_BB919E3D0	Cysteine desulfurase 1, chloroplastic			x
Traes_4AL_45E27280D	Cysteine desulfurase 1, chloroplastic	x		
Traes_3B_3890BB4DE	Cysteine synthase			x
Traes_3AS_A3FC93C65	Cysteine synthase			x
Traes_3B_B8A2DB156	Cysteine synthase			x
Traes_3AL_50A1008AB	Cysteine synthase	x		
Traes_5AS_EAE5D853B	Cysteine synthase	x		
Traes_5BS_1AC8D3009	Cysteine synthase	x		
Traes_7BL_B5491A48C	D-3-phosphoglycerate dehydrogenase			x
Traes_7AL_95C81BBBF	D-3-phosphoglycerate dehydrogenase			x
Traes_1AL_5E4EECFBC	Delta-1-pyrroline-5-carboxylate dehydrogenase 12A1, mitochondrial	x		
Traes_7BS_5781FD481	Dihydroxy-acid dehydratase, chloroplastic			x
Traes_7BS_36E4AE13A	Dihydroxy-acid dehydratase, chloroplastic		x	
Traes_1BL_91B1BCB59	Folylpolyglutamate synthase			x
Traes_4AL_9BCB37710	Glutamate decarboxylase	x		
Traes_4BS_D94FACCE2	Glutamate decarboxylase			x
Traes_3AS_3CB8A9C01	Glutamate decarboxylase 2			x
Traes_2AL_73A59BC32	Glutamate receptor			x
Traes_3AS_31089CA19	Histidine biosynthesis bifunctional protein hisIE, chloroplastic			x
Traes_4AS_E54C88DDB	Imidazole glycerol phosphate synthase hisHF, chloroplastic	x		
Traes_4BL_CF4451CAA	Imidazole glycerol phosphate synthase hisHF, chloroplastic	x		
Traes_6BS_AE8D595A3	L-aspartate oxidase, chloroplastic			x
Traes_2AL_F8F55F0A3	Methionine aminopeptidase			x
Traes_5AL_8349DE248	Ornithine aminotransferase, mitochondrial			x
Traes_5BL_1D3F1BCC2	Ornithine aminotransferase, mitochondrial			x
Traes_6AL_727AEFCD4	Ornithine carbamoyltransferase, chloroplastic	x		
Traes_6BL_0A6B5575F	Ornithine carbamoyltransferase, chloroplastic	x		
Traes_6AS_0C1D497EA	S-adenosylmethionine synthase 4			x

	Traes_6BL_93357D848	Serine/threonine-protein phosphatase			x
	Traes_7AS_F3CC01C3D	Serine--glyoxylate aminotransferase		x	
	Traes_7BL_28E519867	Tryptophan synthase	x		
	Traes_7AL_7C0CB1D06	Tryptophan synthase		x	
Purine/pyrimidine metabolism	Traes_2AL_DB64E18A1	Allantoinase	x		
	Traes_2BL_55D540224	Allantoinase			x
	Traes_6BL_C433D70A3	Dihydropyrimidine dehydrogenase (NADP(+)), chloroplastic		x	
	Traes_5AS_BA57B5B56	Ureidoglycolate hydrolase			x
	Traes_5BS_E660355D6	Ureidoglycolate hydrolase			x
	Traes_3AL_690595C9D	CTP synthase	x		
	Traes_1BL_D633DA4E8	CTP synthase	x		
	Traes_1AL_B68AA4C68	CTP synthase		x	
	Traes_1AL_341E2A7A8	CTP synthase			x
	Traes_1BL_3A901257A	CTP synthase			x
	Traes_3AL_2A172A963	CTP synthase			x
	Traes_5AL_6FB526190	Carbonic anhydrase		x	
	Traes_3AL_CAE12F07B	Phosphotransferase	x		
**Transporter**	**Gene ID**	**Description**	**common**	**Molise Sel. Colli**	**Simeto**
Ammonium transporter	Traes_2AL_CF862CF2D	Ammonium transporter 1 member 4 (AMT1-4)		x	
	Traes_2BL_F1A254464	Ammonium transporter 1 member 4 (AMT1-4)		x	
Nitrate transporter	Traes_7AS_9D057AD42	Protein NRT1/ PTR FAMILY 3.1		x	
Potassium transporter	Traes_1AL_B7757AFDC	Potassium channel AKT2/3		x	
	Traes_5BL_F112FA40E	Potassium transporter	x		
	Traes_7AS_9A01E6B17	Potassium transporter		x	
	Traes_3AL_9E0B84E84	Potassium transporter			x
	Traes_7BS_E3BDDBFEF	Potassium transporter			x

	Traes_5AL_B64648FE6	Putative potassium transporter 12	x		
	Traes_5BL_F43BDBCB9	Putative potassium transporter 12			x
	Traes_5AL_91132361F	K(+) efflux antiporter 5			x
Phosphate transporter	Traes_7AS_735085EFB	Phosphate transporter PHO1 homolog 1			x
Zinc transporter	Traes_2AL_3983FD077	Zinc transporter ZIP			x
Sulfate transporter	Traes_3AL_224FB10D3	Probable sulfate transporter 3.5	x		
Magnesium transporter	Traes_1AL_C04849EE3	Probable magnesium transporter			x
	Traes_3AL_58B8BF68F	Probable magnesium transporter			x
	Traes_4AL_3C35261D2	Magnesium/proton exchanger			x
Sugar transporter	Traes_1AL_039C1D96D	Bidirectional sugar transporter SWEET			x
	Traes_3AL_5A716129C	Bidirectional sugar transporter SWEET			x
	Traes_3AL_729C392FB	Bidirectional sugar transporter SWEET	x		
	Traes_4AS_6D625D7BF	Bidirectional sugar transporter SWEET	x		
	Traes_6AL_58B6B7319	Bidirectional sugar transporter SWEET			x
	Traes_6BL_BE49FDA0B	Bidirectional sugar transporter SWEET			x
	Traes_4AS_A322DBCB0	Sucrose transporter SUT1A	x		
	Traes_4AS_C4001163C	GDP-mannose transporter GONST3			x
Calcium transporter	Traes_1BL_9772C6F45	Calcium-transporting ATPase			x
	Traes_2BL_90036AF45	Calcium-transporting ATPase	x		
	Traes_2BL_E2DCFA60B	Calcium-transporting ATPase		x	
	Traes_4AL_8CAEF3C85	Calcium-transporting ATPase		x	
	Traes_5AL_FA3033A40	Calcium-transporting ATPase			x
	Traes_6AS_9B913DF6F	Calcium-transporting ATPase		x	
	Traes_1BL_9772C6F45	Calcium-transporting ATPase			x
	Traes_5AL_FA3033A40	Calcium-transporting ATPase			x

Sodium transporter	Traes_3AL_9F1E0BAE1	Sodium/pyruvate cotransporter BASS2, chloroplastic		x	
Copper transporter	Traes_2AL_D0EABF355	Probable copper-transporting ATPase HMA5			x
	Traes_2BL_19B3E60AA	Probable copper-transporting ATPase HMA5			x
ABC transporter	Traes_3AS_BB185F2EB	ABC transporter G family member 7	x		
	Traes_3B_5FDDEC00B	ABC transporter G family member 32		x	
	Traes_5AS_C606A8A1F	PDR-type ABC transporter			x
	Traes_3B_77C6EE076	ABC transporter G family member 7			x
	Traes_4AS_6A57D15D9	ABC transporter I family member 6, chloroplastic			x
	Traes_3AS_E18F065A3	Protein ABCI7, chloroplastic			x
Amino acid transporter	Traes_6AL_1E68C6606	Cationic amino acid transporter 9, chloroplastic	x		
	Traes_4AL_1B3E7B791	WAT1-related protein		x	
	Traes_5BL_CBB9D7E7F	WAT1-related protein			x
Other transporter	Traes_2AL_2612F72B9	3-oxoacyl-[acyl-carrier-protein] synthase	x		
	Traes_2AL_341C03FBF	3-oxoacyl-[acyl-carrier-protein] synthase	x		
	Traes_2AL_5745581C6	3-oxoacyl-[acyl-carrier-protein] synthase	x		
	Traes_1BS_8C10A5D1D	Acyl carrier protein (ACP)			x
	Traes_5AL_D9BFBD63A	Acyl carrier protein (ACP)	x		
	Traes_5BL_E9C760122	Acyl carrier protein (ACP)	x		
	Traes_5AS_178DFC4E3	Acyl carrier protein (ACP)	x		
	Traes_5BS_4C0AD2649	Acyl carrier protein (ACP)	x		
	Traes_2AL_E80950527	Chloride channel protein			x
	Traes_5BL_B8C3AE4A5	Chloride channel protein			x
	Traes_6AS_0F6151540	Chloride channel protein			x
	Traes_6AS_28752C23C	Chloride channel protein			x
	Traes_2BL_1356B2300	Chloride channel protein	x		
	Traes_4AL_6240D6D66	Folate transporter 1, chloroplastic			x

Traes_2BL_E2C38B73C	Gamma-soluble NSF attachment protein			x
Traes_6BL_2B67C5E30	Glycolipid transfer protein 1			x
Traes_6BL_2B67C5E30	Glycolipid transfer protein 1			x
Traes_7BL_C46BC291C	Heavy metal transporting P1B-ATPase 2		x	
Traes_7AL_8304348B7	Heavy metal transporting P1B-ATPase 2	x		
Traes_5BL_68C95B396	Mitochondrial import inner membrane translocase subunit TIM10		x	
Traes_1BS_0A858EB86	Mitochondrial pyruvate carrier			x
Traes_1BS_0A858EB86	Mitochondrial pyruvate carrier			x
Traes_4AS_5828A4E4D	Peroxisomal nicotinamide adenine dinucleotide carrier			x
Traes_2AS_F3FE36E3E	Phospholipid-transporting ATPase			x
Traes_2BS_2317C066E	Phospholipid-transporting ATPase			x
Traes_4AS_D950EBDB0	Phospholipid-transporting ATPase			x
Traes_4BL_CA03BD203	Phospholipid-transporting ATPase			x
Traes_4AL_D7702A458	Plasma membrane ATPase			x
Traes_5BS_7DDBE5B89	Plasma membrane ATPase		x	
Traes_4AL_D7702A458	Plasma membrane ATPase			x
Traes_1AL_C544E6828	Plastidal glycolate/glycerate translocator 1, chloroplastic			x
Traes_2AL_E7D1C8AA5	Probable inositol transporter 2			x
Traes_2BL_DE4FAAC5E	Probable inositol transporter 2			x
Traes_2AL_7F21D24BA	Protein TIC 40, chloroplastic			x
Traes_2BL_A4FF3C90C	Protein TIC 40, chloroplastic			x
Traes_5BL_28CCE5325	Protein TIC 62, chloroplastic			x
Traes_7BL_95C7C23DD	Sec-independent protein translocase protein TATB, chloroplastic		x	
Traes_1BL_5F74C58ED	Secretory carrier-associated membrane protein (SCAMPs)		x	
Traes_1AL_090571678	Secretory carrier-associated membrane protein (SCAMPs)		x	
Traes_3AL_697247353	Secretory carrier-associated membrane protein (SCAMPs)			x
Traes_7AS_D1A84E231	Trigger factor-like protein TIG, Chloroplastic	x		
Traes_7BS_B18D7717E	Trigger factor-like protein TIG, Chloroplastic	x		
Traes_5BL_2C3037F54	Vacuolar protein sorting-associated protein 2 homolog 2	x		
Traes_6AL_2C61320D2	Photosynthetic NDH subunit of lumenal location 4, chloroplastic		x	

	Traes_4BL_F37C90CB8	Photosynthetic NDH subunit of subcomplex B 2, chloroplastic		x	

**C metabolism**	**Gene ID**	**Description**	**common**	**Molise Sel. Colli**	**Simeto**
Glycolisis	Traes_2AS_D9D9A9ADB	[Pyruvate dehydrogenase (acetyl-transferring)] kinase, mitochondrial		x	
	Traes_6BS_C9B216D97	Acetyltransferase component of pyruvate dehydrogenase complex		x	
	Traes_2AL_2469A32A8	Aldose 1-epimerase			x
	Traes_2AL_CA75501A81	Aldose 1-epimerase			x
	Traes_2AL_729CB1040	Aldose 1-epimerase			x
	Traes_5AS_C7C69D278	ATP-dependent 6-phosphofructokinase	X		
	Traes_4AL_EBD5A433B	ATP-dependent 6-phosphofructokinase (PFK1)		x	
	Traes_4AL_EBD5A433B	ATP-dependent 6-phosphofructokinase (PFK1)		x	
	Traes_5BL_DECE49DFC	Dihydrolipoamide acetyltransferase component of pyruvate dehydrogenase complex			x
	Traes_5AL_17458C47B	Dihydrolipoamide acetyltransferase component of pyruvate dehydrogenase complex			x
	Traes_5AS_3B966EA46	Dihydrolipoamide acetyltransferase component of pyruvate dehydrogenase complex	x		
	Traes_5AL_32B5C730F	Dihydrolipoamide acetyltransferase component of pyruvate dehydrogenase complex	x		
	Traes_5BL_BA19E1CE3	Dihydrolipoamide acetyltransferase component of pyruvate dehydrogenase complex	x		
	Traes_1BS_15C828137	Dihydrolipoyl dehydrogenase			x
	Traes_5AL_AB5831D39	Enolase 1, chloroplastic		x	
	Traes_5BL_B4537ED84	Enolase 1, chloroplastic			x
	Traes_4BS_D12DBE6D3	Fructose-bisphosphate aldolase			x
	Traes_3AL_441C0AE1B	Fructose-bisphosphate aldolase			x
	Traes_5BS_017F8702A	Fructose-bisphosphate aldolase			x
	Traes_4BS_4404098F1	Fructose-1,6-bisphosphatase, chloroplastic			x
	Traes_2AL_783CF383F	Glyceraldehyde-3-phosphate dehydrogenase			x
	Traes_2BL_5D64E8C87	Glyceraldehyde-3-phosphate dehydrogenase			x
	Traes_4BL_F32809B15	Glyceraldehyde-3-phosphate dehydrogenase			x
	Traes_4BL_A9FAA75A9	Glyceraldehyde-3-phosphate dehydrogenase			x

	Traes_2AL_783CF383F	Glyceraldehyde-3-phosphate dehydrogenase			x
	Traes_2BL_5D64E8C87	Glyceraldehyde-3-phosphate dehydrogenase			x
	Traes_4BL_F32809B15	Glyceraldehyde-3-phosphate dehydrogenase			x
	Traes_4BL_A9FAA75A9	Glyceraldehyde-3-phosphate dehydrogenase			x
	Traes_1BL_F7884B670	Phosphoglycerate kinase (PGK)			x
	Traes_1BL_F7884B670	Phosphoglycerate kinase (PGK)			x
	Traes_2BS_167A31F21	Plastidial pyruvate kinase 1, chloroplastic			x
	Traes_3B_631CEF092	Plastidial pyruvate kinase 2			x
	Traes_3AL_81435CD25	Plastidial pyruvate kinase 2	x		
	Traes_5AS_4C84934FE	Pyrophosphate--fructose 6-phosphate 1-phosphotransferase subunit alpha		x	
	Traes_7AS_25F9FEF52	Pyruvate dehydrogenase E1 component subunit alpha		x	
	Traes_2AS_F485758C1	Pyruvate dehydrogenase E1 component subunit alpha-3, chloroplastic	x		
	Traes_2BL_9B9195C2E	Pyruvate kinase			x
	Traes_5AL_89F608111	Pyruvate kinase			x
	Traes_5BL_6E109E397	Pyruvate kinase			x
	Traes_2AL_D6D188AA7	Pyruvate kinase			x
	Traes_2BL_9B9195C2E	Pyruvate kinase			x
	Traes_5AL_89F608111	Pyruvate kinase			x
	Traes_5BL_6E109E397	Pyruvate kinase			x
	Traes_2AL_D6D188AA7	Pyruvate kinase			x
	Traes_1AL_E5E41283E	Pyruvate, phosphate dikinase 1, chloroplastic	x		
	Traes_1BL_AAC4D956E	Pyruvate, phosphate dikinase 1, chloroplastic	x		
Pentose phosphate shunt	Traes_2AS_67222868A	Glucose-6-phosphate 1-dehydrogenase	x		
	Traes_5AL_C14B0076E	Probable ribose-5-phosphate isomerase 4, chloroplastic			x
	Traes_5BL_063A12847	Probable ribose-5-phosphate isomerase 4, chloroplastic			x
Tricarboxilic acid cycle (TCA)	Traes_2BL_CEC7F2D7B	Isocitrate dehydrogenase [NADP]			x
	Traes_2AL_3668C746C	D-2-hydroxyglutarate dehydrogenase, mitochondrial			x
	Traes_2BL_A42EAB22C	D-2-hydroxyglutarate dehydrogenase, mitochondrial			x

Photosynthesis	Traes_5AL_7E134CAB9	[Fructose-bisphosphate aldolase]-lysine N-methyltransferase, chloroplastic			x
	Traes_5BL_3CD624CE9	[Fructose-bisphosphate aldolase]-lysine N-methyltransferase, chloroplastic			x
	Traes_4AL_70D393C3B	2-carboxy-1,4-naphthoquinone phytyltransferase, chloroplastic	x		
	Traes_2BS_4AE914BE2	Chlorophyll a-b binding protein, chloroplastic			x
	Traes_5BL_2C06592B7	Chlorophyll a-b binding protein, chloroplastic			x
	Traes_5BL_5081CE9EC	Chlorophyll a-b binding protein, chloroplastic			x
	Traes_7AS_C05C7D06B1	Chlorophyll a-b binding protein, chloroplastic			x
	Traes_7BS_91EA4C6E5	Chlorophyll a-b binding protein, chloroplastic			x
	Traes_2BS_F6A5E8996	Chlorophyll a-b binding protein, chloroplastic			x
	Traes_5AL_75780E5B0	Chlorophyll a-b binding protein, chloroplastic		x	
	Traes_1AL_053F6B12B	Chlorophyll synthase, chloroplastic	x		
	Traes_1AL_1A98A80B4	Chlorophyll synthase, chloroplastic			x
	Traes_6AS_E114D6D5B	Delta-aminolevulinic acid dehydratase	x		
	Traes_6BS_0726A0A88	Delta-aminolevulinic acid dehydratase	x		
	Traes_6AS_F205EE33F	Delta-aminolevulinic acid dehydratase	x		
	Traes_7AL_3C614133C	Delta-aminolevulinic acid dehydratase	x		
	Traes_7BL_C35CD97E7	Delta-aminolevulinic acid dehydratase	x		
	Traes_7AL_7C99238B0	Ferredoxin			x
	Traes_2BL_363CA4ED8	Ferredoxin-3			x
	Traes_2AL_688678052	Ferredoxin-3			x
	Traes_5AL_B0E91B780	Ferredoxin--NADP reductase	x		
	Traes_5AL_B0E91B780	Ferredoxin--NADP reductase	x		
	Traes_5AL_23B24917C	Ferrochelatase			x
	Traes_5BL_FF9AA4B58	Ferrochelatase			x
	Traes_5AL_3EED2020C	Magnesium-chelatase subunit ChlD, chloroplastic			x
	Traes_5AL_F490CE585	Magnesium-chelatase subunit ChlD, chloroplastic	x		
	Traes_5BL_1DE088A30	Magnesium-chelatase subunit ChlD, chloroplastic	x		
	Traes_5BL_CFC7BB483	Magnesium-chelatase subunit ChlD, chloroplastic	x		
	Traes_7BL_82850CC15	Mg-protoporphyrin IX chelatase			x

	Traes_7AL_8481A6FD7	Mg-protoporphyrin IX chelatase	x		
	Traes_7BL_12EBCA739	Mg-protoporphyrin IX chelatase	x		
	Traes_3AL_6BDD6F090	NAD(P)H-quinone oxidoreductase subunit O, chloroplastic			x
	Traes_5AL_3C4AACD91	Probable zinc metalloprotease EGY1, chloroplastic	x		
	Traes_3AS_922982272	Probable zinc metalloprotease EGY2	x		
	Traes_5AS_3354E01BA	Protoporphyrinogen oxidase 1, chloroplastic (PPOX1)	x		
	Traes_5BS_5C3BA5328	Protoporphyrinogen oxidase 1, chloroplastic (PPOX1)	x		
	Traes_2AL_1AD2DDF83	Protoporphyrinogen oxidase 2, chloroplastic/mitochondrial			x
	Traes_5BS_69F251543	PsbP domain-containing protein 1, chloroplastic	X		
	Traes_2BL_ACD272701	PsbP domain-containing protein 2, chloroplastic	X		
	Traes_2AL_57B2ACCC0	PsbP domain-containing protein 2, chloroplastic			x
	Traes_6AS_FD6AE4DD4	Red chlorophyll catabolite reductase, chloroplastic			x
	Traes_2AS_2B3603918	Ribulose bisphosphate carboxylase small chain (RUBISCO)			x
	Traes_2AS_C3EF3A27F	Ribulose bisphosphate carboxylase small chain (RUBISCO)			x
	Traes_2AS_DDDAF2E70	Ribulose bisphosphate carboxylase small chain (RUBISCO)			x
	Traes_2BS_0AF61748C	Ribulose bisphosphate carboxylase small chain (RUBISCO)			x
	Traes_2BS_E68F1B47C	Ribulose bisphosphate carboxylase small chain (RUBISCO)			x
	Traes_2AS_7425ED39F	Ribulose bisphosphate carboxylase small chain (RUBISCO)			x
	Traes_3AL_D2C3599B4	Uroporphyrinogen decarboxylase (UROD)	x		
	Traes_4BL_B902E85F5	Uroporphyrinogen decarboxylase 2, chloroplastic (UROD2)	x		
	Traes_4AS_90CC29CAA	Uroporphyrinogen decarboxylase 2, chloroplastic (UROD2)			x
Photorespiration	Traes_7AS_95F4E4729	Carboxyl-terminal-processing peptidase 3, chloroplastic	x		
	Traes_7BS_462DD3C32	Carboxyl-terminal-processing peptidase 3, chloroplastic	x		
	Traes_7BS_7E9C070B8	Carboxyl-terminal-processing peptidase 3, chloroplastic	x		
	Traes_1AL_C544E6828	Plastidal glycolate/glycerate translocator 1, chloroplastic			x
Fatty acids metabolism	Traes_4AS_E1B651375	3-ketoacyl-CoA synthase		x	
	Traes_4BL_A71648154	3-ketoacyl-CoA synthase 10			x
	Traes_4AS_D58530D14	3-ketoacyl-CoA synthase 10			x
	Traes_5AL_4ACF1AEBB	Acyl-[acyl-carrier-protein] desaturase	x		
	Traes_5BL_3CEA3D2B4	Acyl-[acyl-carrier-protein] hydrolase		x	

	Traes_5AL_CEEDF43FC	Acyl-[acyl-carrier-protein] hydrolase		x	
	Traes_3AS_0EA847466	Fatty acyl-CoA reductase			x
	Traes_5AL_DC104F3FE	Fatty acyl-CoA reductase			x
	Traes_7BS_2DA5DB033	Fatty acyl-CoA reductase			x
	Traes_4BS_DA955986B	Fatty acyl-CoA reductase			x
	Traes_4BS_DA955986B	Fatty acyl-CoA reductase			x
	Traes_6AL_0C5F4D84E	Fatty-acid-binding protein 2			x
	Traes_2AL_F1C4FF20C	Lecithin-cholesterol acyltransferase-like 4	x		
	Traes_2BL_3DBAA0726	Lecithin-cholesterol acyltransferase-like 4			x
	Traes_2BL_3DBAA0726	Lecithin-cholesterol acyltransferase-like 4			x
	Traes_1AS_522839777	Long chain acyl-CoA synthetase 1 (LCAS 1)	x		
	Traes_1AS_0EAA8A0B4	Long chain acyl-CoA synthetase 1		x	
	Traes_1BS_3C86AE038	Long chain acyl-CoA synthetase 1		x	
	Traes_5AL_3803F504A	Long chain acyl-CoA synthetase 8			x
	Traes_2AS_2980E0300	Omega-6 fatty acid desaturase, chloroplastic			x
	Traes_4AS_D2A85E4CA	Patatin		x	
	Traes_7AL_CB50E0F46	Patatin	x		
	Traes_2BL_D1BE1544E	Protein S-acyltransferase 21		x	
	Traes_6BL_D1538B2A7	Very-long-chain (3R)-3-hydroxyacyl-CoA dehydratase			x
Other metabolism	Traes_7AL_1E9FBDD47	1,4-alpha-glucan-branching enzyme 3, chloroplastic/amyloplastic	x		
	Traes_7AL_5D8D7CB60	1,4-alpha-glucan-branching enzyme 3, chloroplastic/amyloplastic			x
	Traes_3AL_A3B273BAC	1,4-dihydroxy-2-naphthoyl-CoA synthase, peroxisomal			x
	Traes_2AL_73EA196D5	2-phytyl-1,4-beta-naphthoquinone methyltransferase, chloroplastic			x
	Traes_3AS_6F9355B92	3-epi-6-deoxocathasterone 23-monooxygenase			x
	Traes_2BS_8D01B66C0	4-alpha-glucanotransferase DPE1, chloroplastic/amyloplastic (maltotriose metabolism)	x		
	Traes_2AS_3C5CB1C2C	4-alpha-glucanotransferase DPE1, chloroplastic/amyloplastic (maltotriose metabolism)	x		
	Traes_3AL_8C6F4F66A	4-diphosphocytidyl-2-C-methyl-D-erythritol kinase, chloroplastic	x		

Traes_4AS_80A9FCCF4	Alpha-1,3-glucosyltransferase			x
Traes_1AL_ED72E167A	Alpha-galactosidase		x	
Traes_2AS_65297DABA	Alpha-galactosidase 3	x		
Traes_5AL_ED21C722B	Alpha-glucan phosphorylase 1		x	
Traes_5AL_D86A878AF	Alpha-mannosidase	x		
Traes_5BL_BF60CBEDA	Alpha-mannosidase	x		
Traes_2AS_E0AFA6D18	ATP sulfurylase 2			x
Traes_2BS_F70FE410B	ATP sulfurylase 2			x
Traes_3B_55C9357A4	Beta-amylase			x
Traes_2AS_61CA61AD3	Beta-amylase	x		
Traes_2BS_A1909D302	Beta-amylase	x		
Traes_7AL_AFC2A9D5D	Beta-1,6-galactosyltransferase GALT31A		x	
Traes_4AS_4C25DF6F4	Beta-galactosidase		x	
Traes_4AS_BB59116B6	Beta-galactosidase 8		x	
Traes_1BL_C955569B8	Beta-hexosaminidase	x		
Traes_1BS_014C64CC2	Beta-hexosaminidase 1		x	
Traes_1AL_8E8E7401B	Beta-hexosaminidase		x	
Traes_5AL_05D58A0C4	Cellulose synthase A catalytic subunit 7			x
Traes_3AL_FFE4C92D8	Cellulose synthase A catalytic subunit 8 [UDP-forming]			x
Traes_5BL_E86097AA2	Chalcone-flavonone isomerase family protein			x
Traes_6BL_3C04C3D2F	Chlorophyll(ide) b reductase NOL, chloroplastic			x
Traes_6BL_3C04C3D2F	Chlorophyll(ide) b reductase NOL, chloroplastic			x
Traes_5BS_012B7F86A	Cyclic pyranopterin monophosphate synthase, mitochondrial			x
Traes_4AL_BB919E3D0	Cysteine desulfurase 1, chloroplastic			x
Traes_3AL_883A4805C	Cytokinin riboside 5'-monophosphate phosphoribohydrolase		x	
Traes_7BL_EA8DE756D	Flavin-containing monooxygenase			x
Traes_2AL_06EF31003	Galacturonokinase		x	
Traes_2BL_4DDAB77A8	Galacturonokinase		x	
Traes_7BS_4FBE4B00A	Glucose-1-phosphate adenylyltransferase	x		
Traes_3AL_F3125B233	Glycerol-3-phosphate dehydrogenase [NAD(+)] 1, chloroplastic		x	
Traes_3AL_12CDB0C85	Glycerol-3-phosphate dehydrogenase [NAD(+)]		x	

Traes_3AL_30294D0A5	Glycerol-3-phosphate dehydrogenase [NAD(+)]		x	
Traes_6AS_626A3D38C	Glycosyltransferase		x	
Traes_5BL_BD269CC23	Glycosyltransferase			x
Traes_3AL_829F70144	Glycosyltransferase	x		
Traes_3AL_A0C2D775D	Glycosyltransferase	x		
Traes_5BL_406A1C48D	Glycosyltransferase			x
Traes_2AL_06AD35739	Granule-bound starch synthase 1, chloroplastic/amyloplastic			x
Traes_2BL_25DAD57C9	Granule-bound starch synthase 1, chloroplastic/amyloplastic			x
Traes_2BL_4470DF166	Granule-bound starch synthase 1, chloroplastic/amyloplastic			x
Traes_7AL_3F7F0AA7B	Granule-bound starch synthase 1, chloroplastic/amyloplastic			x
Traes_2AL_BE5540B1E	Malate synthase (pyruvate metabolism)			x
Traes_3AL_C0D1C4403	Malic enzyme (pyruvate metabolism)			x
Traes_5AL_C1E3FCB4F	Monogalactosyldiacylglycerol synthase 1, chloroplastic			x
Traes_2AS_DEDC612AE	Nicotianamine synthase		x	
Traes_5BS_E7ADA47A4	Pectinesterase	x		
Traes_1AL_E9BA105D1	Pectinesterase 31			x
Traes_5AS_F24765BD1	Pectinesterase		x	
Traes_4BL_2465F30E0	Pheophorbide a oxygenase, chloroplastic	x		
Traes_4BL_4447E55C5	Pheophorbide a oxygenase, chloroplastic	x		
Traes_7AL_72A908F12	Pheophytinase, chloroplastic	x		
Traes_1BS_4A63D1DBC	Phosphatidate cytidylyltransferase			x
Traes_1AS_9D4506E73	Phosphatidate cytidylyltransferase			x
Traes_1BS_4A63D1DBC	Phosphatidate cytidylyltransferase			x
Traes_1AS_9D4506E73	Phosphatidate cytidylyltransferase			x
Traes_5AL_636F300F6	Phosphoglucan phosphatase LSF2, chloroplastic	x		
Traes_2AL_81CAF6C30	Phosphomannomutase	x		
Traes_4BL_D08D5FFB5	Phosphomannomutase	x		
Traes_2BL_EB792A35E	Phosphomannomutase			x
Traes_6BS_A80520954	Phosphopantetheine adenylyltransferase			x
Traes_2AL_62160B4F7	Probable alpha-amylase 2			x
Traes_7AL_6A30E603B	Probable galacturonosyltransferase 3		x	
Traes_7BS_092F4241C	Pullulanase 1, chloroplastic		x	

	Traes_5AS_7E93CEA52	Purple acid phosphatase (PAP)		x	
	Traes_5BL_6D2126519	Purple acid phosphatase (PAP)	x		
	Traes_5AS_D42C83AB8	Purple acid phosphatase (PAP)			x
	Traes_4BL_840A609C1	Purple acid phosphatase (PAP)			x
	Traes_4BL_598C0826B	Purple acid phosphatase (PAP)			x
	Traes_4BL_99574CDAC	Purple acid phosphatase (PAP)			x
	Traes_5AL_394CDB9BC	Purple acid phosphatase (PAP)			x
	Traes_7AL_9BC56072C	Putative beta-glucosidase 41			x
	Traes_7AL_AA28D70BF	Starch branching enzyme I	x		
	Traes_7AS_A80BE362A	Sucrose synthase		x	
	Traes_4AL_FE0C5AEF4	Sucrose:fructan 6-fructosyltransferase			x
	Traes_2BS_8800ED4EE	Terpene cyclase/mutase family member			x
	Traes_7AS_E82D29D97	Terpene cyclase/mutase family member			x
	Traes_6AS_F3CC61A36	Terpene cyclase/mutase family member		x	
	Traes_1AL_0FB80B128	Uracil phosphoribosyltransferase, chloroplastic	x		
	Traes_6AL_006779D22	Xyloglucan endotransglucosylase/hydrolase		x	
	Traes_4BS_64FB912EE	Xyloglucan endotransglucosylase/hydrolase	x		
	Traes_7AL_1B1FBCDE4	Xyloglucan endotransglucosylase/hydrolase (XTHs)			x
	Traes_4AL_320703FD2	Xyloglucan endotransglucosylase/hydrolase (XTHs)			x
	Traes_7AL_8C4A9BEBF	Xyloglucan endotransglucosylase/hydrolase (XTHs)			x
	Traes_2BS_D4E86AAA6	Xylose isomerase		x	
	Traes_7AL_AFA2C29C9	Photosystem II stability/assembly factor HCF136, chloroplastic			x
	Traes_7BL_CA98655DC	Photosystem II stability/assembly factor HCF136, chloroplastic			x
**Transcription factors**	**Gene ID**	**Description**	**common**	**Molise Sel. Colli**	**Simeto**
	Traes_3AL_7A2CED8E7	Auxin response factor (ARFs Family)	x		
	Traes_3AL_E34DF2F08	Auxin response factor (ARFs Family)	x		
	Traes_3AL_5935773EA	Auxin response factor (ARFs Family)			x
	Traes_1BL_A9AC1340E	Auxin response factor (ARFs Family)			x
	Traes_1AL_147CF243C	Auxin response factor (ARFs Family)			x
	Traes_5BL_C86AC392A	Auxin-responsive protein		x	

Traes_5BL_9301BD154	Auxin-responsive protein			x
Traes_5BL_EC006AD0C	Auxin-responsive protein			x
Traes_4BL_7DA93696E	CCAAT-binding transcription factor B (NF-Y family)	x		
Traes_6AL_C0E74F959	CCAAT-binding transcription factor B (NF-Y family)	x		
Traes_6BL_953171851	CCAAT-binding transcription factor B (NF-Y family)	x		
Traes_5AL_3270A6690	Enhanced ethylene response protein 5			x
Traes_5BL_7F84602F3	Ethylene responsive transcription factor 5a			x
Traes_7BS_B1A8B5B8A	Ethylene-responsive element binding protein 1			x
Traes_2BL_94E5996F7	FD-like 15 protein			x
Traes_7BL_EA8DE756D	Flavin-containing monooxygenase			x
Traes_6AS_2B85F4104	Mediator of RNA polymerase II transcription subunit 18			x
Traes_5AL_13E2DEC48	MIKC-type MADS-box transcription factor WM6			x
Traes_4AL_98EA87DFA1	MYB-related protein (MYB family)			x
Traes_4BL_4D370C9FA	Probable mediator of RNA polymerase II transcription subunit 37c		x	
Traes_4BL_4D370C9FA1	Probable mediator of RNA polymerase II transcription subunit 37c		x	
Traes_3AS_E18F065A3	Protein ABCI7, chloroplastic			x
Traes_3AL_E5CB6F48A	Protein PLASTID TRANSCRIPTIONALLY ACTIVE 12 (PTAC family)			x
Traes_3AL_89EF640CE	Protein PLASTID TRANSCRIPTIONALLY ACTIVE 12 (PTAC family)	x		
Traes_1AL_D8BA4FB5D	Protein PLASTID TRANSCRIPTIONALLY ACTIVE 14 (PTAC family)	x		
Traes_1BL_E1AD16CAC	Protein PLASTID TRANSCRIPTIONALLY ACTIVE 14 (PTAC family)	x		
Traes_3AL_C25CC8AB1	Protein PLASTID TRANSCRIPTIONALLY ACTIVE 7 (PTAC family)	x		
Traes_2AL_5BA7E2623	TaAP2-A			x
Traes_7AL_6279FCE8B	Transcription activator GLK2			x
Traes_7AL_6279FCE8B	Transcription activator GLK2			x
Traes_6BS_05264DAEA	Transcription factor HBP-1a			x
Traes_1BL_AF69F4610	Transcription factor UNE10		x	
Traes_1AL_914726769	Transcription factor UNE10		x	
Traes_7AS_3BAA88E9E	Transcription termination factor MTERF6,	x		

		chloroplastic/mitochondrial			
	Traes_3B_07317CB79	Transcriptional corepressor LEUNIG		x	
	Traes_4AL_26117D669	RNA polymerase sigma factor	x		
	Traes_4AS_26117D669	RNA polymerase sigma factor	x		
	Traes_4AS_DE644E0CF	RNA polymerase sigma factor			x
	Traes_7AS_3BAA88E9E	Transcription termination factor MTERF6, chloroplastic/mitochondrial	x		
	Traes_2AS_94603BB74	Translation initiation factor	x		
**Kinases**	**Gene ID**	**Description**	**common**	**Molise Sel. Colli**	**Simeto**
	Traes_4AS_A795672BE	Adenylyl-sulfate kinase	x		
	Traes_1AL_928DC3A3C	Bifunctional riboflavin kinase/FMN phosphatase (FHY)	x		
	Traes_1AL_7C3E4A07E	Diacylglycerol kinase (DGK)			x
	Traes_5AS_C28641518	Diacylglycerol kinase (DGK)			x
	Traes_3AL_3BC2FF0ED	Fructokinase-like 1, chloroplastic			x
	Traes_2BS_959A4E58A	Fructokinase-like 2, chloroplastic	x		
	Traes_4AL_8C4927CF5	Lectin receptor kinase		x	
	Traes_6AS_691D1AC82	Non-specific serine/threonine protein kinase		x	
	Traes_1AL_5F0F4FAFB	Non-specific serine/threonine protein kinase		x	
	Traes_4AS_538F2E3BD	Non-specific serine/threonine protein kinase			x
	Traes_1BS_C385DE03B	Non-specific serine/threonine protein kinase	x		
	Traes_2BS_0ABED462E	Non-specific serine/threonine protein kinase	x		
	Traes_3AL_66EAB4B4F	Non-specific serine/threonine protein kinase	x		
	Traes_5BL_12214988F	Non-specific serine/threonine protein kinase	x		
	Traes_2AS_6345FCE27	Non-specific serine/threonine protein kinase			x
	Traes_1AL_1538AC680	Nucleoside diphosphate kinase (NDK)		x	
	Traes_1BL_ACC9DDA86	Nucleoside diphosphate kinase (NDK)		x	
	Traes_5AS_8A3C47D2F	Nucleoside diphosphate kinase (NDK)	x		
	Traes_1AL_6F2E87864	Nucleoside diphosphate kinase 1	x		
	Traes_7BS_BEA71AF3F	Receptor-like serine/threonine protein kinase 2	x		
	Traes_6BS_C44E33CC9	Receptor-like serine/threonine-protein kinase NCRK			x
	Traes_3AL_DBED56B67	Serine/threonine-protein kinase	x		

	Traes_5BL_DFFA40472	Serine/threonine-protein kinase	x		
	Traes_2BS_EA063BB91	Serine/threonine-protein kinase		x	
	Traes_2AL_BDC8DD6C7	Serine/threonine-protein kinase			x
	Traes_2BL_07796B6EA	Serine/threonine-protein kinase			x
	Traes_2BL_6FB5FEFC61	Serine/threonine-protein kinase			x
	Traes_2BL_9B20E7E7D	Serine/threonine-protein kinase			x
	Traes_6AL_7D22244E7	Serine/threonine-protein kinase			x
	Traes_2BL_09098E11D	Serine/threonine-protein kinase			x
	Traes_2BL_E1C37619B	Serine/threonine-protein kinase			x
	Traes_5AL_BC7DEEA21	Serine/threonine-protein kinase TOUSLED			x
	Traes_2BL_D195BD715	Serine/threonine-protein kinase			x
	Traes_7AS_7F2987981	Uridine kinase	x		
	Traes_7BS_C57477AA31	Uridine kinase			x

**Stress**	**Gene ID**	**Description**	**common**	**Molise Sel. Colli**	**Simeto**
	Traes_6BL_4DB266957	2-C-methyl-D-erythritol 2,4-cyclodiphosphate synthase, chloroplastic		x	
	Traes_7AL_772A49098	2-succinylbenzoate--CoA ligase, chloroplastic/peroxisomal	x		
	Traes_7AS_C73647B58	3-hydroxy-3-methylglutaryl coenzyme A reductase	x		
	Traes_2BL_657D93794	6,7-dimethyl-8-ribityllumazine synthase (riboflavin synthase)	x		
	Traes_1AS_BEFEF1613	Acetylornithine aminotransferase, chloroplastic/mitochondrial			x
	Traes_2AL_CD28AB70E	Amine oxidase			x
	Traes_2AL_F4F6F518D	Amine oxidase			x
	Traes_2BS_65836FEE4	Amine oxidase	x		
	Traes_2AS_E0AFA6D18	ATP sulfurylase 2			x
	Traes_2BS_F70FE410B	ATP sulfurylase 2			x
	Traes_1AL_52E56C8D3	ATP synthase subunit beta		x	
	Traes_3B_00CF2A894	ATP-dependent Clp protease proteolytic subunit 3, chloroplastic			x
	Traes_4AS_0B56CEBD5	ATP-dependent Clp protease proteolytic subunit-related protein 3, chloroplastic	x		
	Traes_2AL_1E96BBDD4	Autophagy-related protein 101			x

Traes_3AL_27DCCC109	Autophagy-related protein 7c			x
Traes_3AL_81D411955	Autophagy-related protein 7c			x
Traes_5BL_763F3FEA8	Autophagy-related protein 8i			x
Traes_7BL_6097E6ADE	Beta-carotene isomerase D27, chloroplastic			x
Traes_5BL_F0FC14B2A	Bifunctional monothiol glutaredoxin-S16, chloroplastic			x
Traes_7BS_8B18BE79B	Caleosin	x		
Traes_6AS_FC9E9843D	Caleosin	x		
Traes_4BL_53CBCD014	Calmodulin TaCaM1-1	x		
Traes_2BS_D4CB3FB08	Carboxypeptidase			x
Traes_3AS_B605964C1	Carboxypeptidase			x
Traes_1AL_16EAFFACD	Carboxypeptidase			x
Traes_4BS_AD3C7F651	Carboxypeptidase		x	
Traes_1BS_FEA181281	Carboxypeptidase		x	
Traes_3AL_A306585EF	Carboxypeptidase	x		
Traes_1AL_B55CC2568	Carotene epsilon-monooxygenase, chloroplastic			x
Traes_1BL_714F4E4AC	Carotene epsilon-monooxygenase, chloroplastic			x
Traes_4BL_664A41517	Catalase			x
Traes_5AL_34C62DB80	CDGSH iron-sulfur domain-containing protein NEET			x
Traes_5BL_E86097AA2	Chalcone-flavonone isomerase family protein			x
Traes_3AL_883A4805C	Cytokinin riboside 5'-monophosphate phosphoribohydrolase		x	
Traes_7BS_5781FD481	Dihydroxy-acid dehydratase, chloroplastic			x
Traes_5AL_288D3A2F4	Diphosphomevalonate decarboxylase			x
Traes_5BL_AC016C8E2	Diphosphomevalonate decarboxylase		x	
Traes_7AL_F516C7DFF	Dolichyl-diphosphooligosaccharide--protein glycosyltransferase subunit DAD1			x
Traes_2AS_7D3EC9FC2	E3 ubiquitin-protein ligase		x	
Traes_7AS_C38093E5C	ECPT-type aminoalcoholphosphotransferase			x
Traes_4AS_94B8E6F8C	FAD-linked sulfhydryl oxidase ERV1			x
Traes_7AS_34AF6845D1	F-box protein MAX2		x	
Traes_7BS_3285CDC24	F-box protein MAX2	x		
Traes_4AL_497775E8F	ferritin			x
Traes_4BS_FFF12BA9E	ferritin			x

Traes_4AL_498512281	ferritin	x		
Traes_4BS_BA655294C	ferritin	x		
Traes_7BL_EA8DE756D	Flavin-containing monooxygenase			x
Traes_7AS_F91D8701E	GA-induced protein			x
Traes_6AS_4C413AC53	Gamma-glutamylcyclotransferase		x	
Traes_6BS_A679AA5FF	Gamma-glutamylcyclotransferase		x	
Traes_1AL_F18EC96DF	GBF1			x
Traes_2AL_E06A248EE	Glutathione peroxidase			x
Traes_4AS_519B67360	Glutathione reductase, chloroplastic	x		
Traes_4BL_9BA5AB82C	Glutathione reductase, chloroplastic	x		
Traes_7AS_0BB13DD79	Glutathione S-transferase DHAR3, chloroplastic			x
Traes_6AS_D537A116B	Glutathione synthetase		x	
Traes_6BS_AA7101C2C	Glutathione synthetase			x
Traes_3AL_1D9B2CE84	Glyoxylate/succinic semialdehyde reductase 2, chloroplastic			x
Traes_2BL_088F3C674	Guard cell S-type anion channel SLAC1			x
Traes_5BL_37ECD3B1E	Heat shock protein 90-5, chloroplastic			x
Traes_5AL_1DA3B4631	Heat shock protein 90-5, chloroplastic		x	
Traes_4AS_53B185E1A	HVA22-like protein	x		
Traes_2BL_7BB7F9970	HVA22-like protein			x
Traes_1BL_7F9006C51	Hyperosmolality-gated Ca2+ permeable channel		x	
Traes_5AL_07C125B1C	Hypersensitive induced reaction protein 2			x
Traes_5AL_C0A407A93	Hypersensitive induced response protein 3			x
Traes_3AL_E5520535A	Laccase			x
Traes_2AS_B2C72994E	Lactoylglutathione lyase			x
Traes_4AL_A5AC2BDF2	Lipoxygenase			x
Traes_2AL_5BAB26827	Lipoxygenase		x	
Traes_5BS_060785740	Lipoxygenase		x	
Traes_2BL_77148B8D8	Lipoxygenase	x		
Traes_5BL_304FAFA26	Lipoxygenase			x
Traes_4BL_BD1E1BEFA	MLO-like protein			x
Traes_2AS_1FC67C1BD	MLO-like protein		x	
Traes_5AL_268DF2E10	MLO-like protein		x	

Traes_5AL_E7A3EDC5F	MLO-like protein		x	
Traes_2AS_9F72E517F	NADPH-dependent thioredoxin reductase 3			x
Traes_6AS_101337AE9	NO-associated protein 1, chloroplastic/mitochondrial		x	
Traes_4BL_96C906D61	Non-specific lipid-transfer protein		x	
Traes_3AS_5A72CA3A7	Non-specific lipid-transfer protein			x
Traes_7BL_2DBEEEBC2	Nudix hydrolase 10	x		
Traes_5AL_43F9E9C1D	Nudix hydrolase 23, chloroplastic			x
Traes_5BL_F0D3D6D2E	Nudix hydrolase 23, chloroplastic	x		
Traes_5AL_64517027B	Obg-like ATPase 1	x		
Traes_5BL_907D7067C	Obg-like ATPase 1	x		
Traes_5AL_4A88E0A3A	Pectin acetylesterase			x
Traes_3AL_11D85AEF9	Pectin acetylesterase			x
Traes_5AL_955D2899A	Pectin acetylesterase			x
Traes_6BL_C83912BDE	Pectin acetylesterase			x
Traes_2AL_6A0320615	Pectin acetylesterase		x	
Traes_1BL_46FB3D95E	Peptide chain release factor PrfB3, chloroplastic			x
Traes_7BL_8930CD1E5	Peptide methionine sulfoxide reductase B1, chloroplastic		x	
Traes_1AS_C70D49E2E	Peroxidase			x
Traes_2AL_20BC426B9	Peroxidase			x
Traes_2AL_2224DCC1A	Peroxidase			x
Traes_2AL_520618712	Peroxidase			x
Traes_7AL_3E96F374E	Peroxidase			x
Traes_7AS_020CCE3DB	Peroxiredoxin Q, chloroplastic			x
Traes_5AL_2F97350D2	Phospholipase D delta	x		
Traes_5BL_A0ED5623C	Phospholipase D delta		x	
Traes_4AS_1D24D36C4	Photosystem II repair protein PSB27-H1, chloroplastic			x
Traes_7AL_E9733B552	Poly [ADP-ribose] polymerase (PARP)			x
Traes_6AS_0F0809F96	Probable inactive linolenate hydroperoxide lyase			x
Traes_1AS_4C784AA40	Protease Do-like 2, chloroplastic	x		
Traes_6BS_4A0C34E63	Protein ACCUMULATION AND REPLICATION OF CHLOROPLASTS 6, chloroplastic	x		
Traes_1BS_8C0F2821A	Protein ALUMINUM SENSITIVE 3		x	

Traes_5AL_5AF616715	Protein C2-DOMAIN ABA-RELATED 11		x	
Traes_2BS_7093A1D1A	Protein DETOXIFICATION			x
Traes_5BL_5A648D29D	Protein DETOXIFICATION		x	
Traes_7AS_85178E0D7	Protein DETOXIFICATION		x	
Traes_3B_189FA2C94	Protein DETOXIFICATION			x
Traes_7AS_E477E850F	Protein DETOXIFICATION			x
Traes_7BS_6A8DBF06C	Protein DETOXIFICATION			x
Traes_5AL_9697E163B	Protein DETOXIFICATION	x		
Traes_4AL_5EC1ABB77	Protein DETOXIFICATION	x		
Traes_7AL_970814850	Protein DETOXIFICATION	x		
Traes_1AL_A970EBB27	Protein DETOXIFICATION 42			x
Traes_1BL_DCC58CE85	Protein DETOXIFICATION 42		x	
Traes_4AS_28DA71FF6	Protein DETOXIFICATION 43			x
Traes_4BL_06F78B23C	Protein DETOXIFICATION 43			x
Traes_6AL_763EE2AED	Protein DETOXIFICATION 45, chloroplastic			x
Traes_1AL_4B7636BC9	Protein LOL1		x	
Traes_7BL_E2E7AA383	Protein OVEREXPRESSOR OF CATIONIC PEROXIDASE 3		x	
Traes_1AL_FE094DCAF	Protein PALE CRESS, chloroplastic			x
Traes_1BL_74FC9D6D1	Protein PALE CRESS, chloroplastic			x
Traes_1AL_AF222553D	Rac-like GTP-binding protein ARAC3			x
Traes_6AL_6CD6A9215	Rhodanese-like domain-containing protein 11, chloroplastic			x
Traes_6BL_F4AF4CD45	Rhodanese-like domain-containing protein 11, chloroplastic			x
Traes_6AS_FD8F6B539	Rhodanese-like domain-containing protein 7	x		
Traes_6AL_E70725BF0	Riboflavin biosynthesis protein PYRR, chloroplastic			x
Traes_6AL_D13D9D951	Serine carboxypeptidase-like 34			x
Traes_4AS_060E22DA0	Serine/arginine-rich splicing factor SR45a		x	
Traes_4BL_7BFE58ED9	Serine/arginine-rich splicing factor SR45a		x	
Traes_4BL_F6A0E1CCB	Signal recognition particle 43 kDa protein, chloroplastic			x
Traes_6AL_5537AC81D	Soluble inorganic pyrophosphatase 6, chloroplastic	x		
Traes_6BL_92B298585	Soluble inorganic pyrophosphatase 6, chloroplastic	x		
Traes_2BL_CCD296233	Stress enhanced protein 2, chloroplastic	x		

	Traes_2AS_0C3C91D16	Stress responsive protein		x	
	Traes_2BS_762E60A0B	Stress responsive protein 1		x	
	Traes_6BS_1C714CE3E	Succinate-semialdehyde dehydrogenase, mitochondrial			x
	Traes_2AL_D0D84176E	Superoxide dismutase	x		
	Traes_7BL_70F07C889	Superoxide dismutase [Cu-Zn] 2, chloroplastic			x
	Traes_7BL_A42D6C984	Superoxide dismutase [Cu-Zn] 2, chloroplastic			x
	Traes_4AL_47DDDE0C1	Thioredoxin-like protein CITRX, chloroplastic			x
	Traes_4BS_D9A4219F2	Thioredoxin-like protein CITRX, chloroplastic		x	
	Traes_6BL_462C2179A	Tripeptidyl-peptidase 2		x	
	Traes_6BL_4DB1AC6CC	Tripeptidyl-peptidase 2		x	
	Traes_1AL_0C964B45F	Tubulin alpha chain		x	
	Traes_2BS_AA6D8150A	Tubulin alpha chain		x	
	Traes_2AS_34272605C	Tubulin alpha chain		x	
	Traes_4BS_0BDB0D05C	Tubulin beta chain			x
	Traes_1BS_2F6DD776E	Tubulin-folding cofactor E			x
	Traes_6AL_4048F89A6	Ubiquitin carboxyl-terminal hydrolase			x
	Traes_3AL_6AF4297B2	Vacuolar proton ATPase subunit E			x
	Traes_2BL_5E868851D	Violaxanthin de-epoxidase, chloroplastic			x
	Traes_2AL_FA3E7C60B	Violaxanthin de-epoxidase, chloroplastic			x
	Traes_5BS_2983EE249	V-type proton ATPase proteolipid subunit			x
	Traes_5BL_2983EE249	V-type proton ATPase proteolipid subunit			x
	Traes_3AL_182FFAF75	Ycf3-interacting protein 1, chloroplastic			x

**Others**	**Gene ID**	**Description**	**common**	**Molise Sel. Colli**	**Simeto**
	Traes_5AL_A8C598914	20 kDa chaperonin, chloroplastic	x		
	Traes_5BL_5041B968E	20 kDa chaperonin, chloroplastic	x		
	Traes_4AS_C420DEB6D	30S ribosomal protein S1, chloroplastic			x
	Traes_4BL_C3C6C11C4	30S ribosomal protein S1, chloroplastic			x
	Traes_4AS_6875248F8	30S ribosomal protein S10, chloroplastic	x		
	Traes_4BL_EFFA96D15	30S ribosomal protein S10, chloroplastic	x		
	Traes_4BS_0ECAB85C2	30S ribosomal protein S13, chloroplastic	x		
	Traes_7AL_A1ECABC74	30S ribosomal protein S31, chloroplastic	x		

Traes_7BL_E67311208	30S ribosomal protein S31, chloroplastic			x
Traes_4AL_FB6127E85	30S ribosomal protein S6 alpha, chloroplastic	x		
Traes_5BL_BB5612CA4	30S ribosomal protein S6 alpha, chloroplastic	x		
Traes_5AL_55D7BFD57	30S ribosomal protein S9, chloroplastic			x
Traes_2AS_105B1A29A	40S ribosomal protein S12	x		
Traes_2BS_7700613D4	40S ribosomal protein S12	x		
Traes_4BS_7700613D4	40S ribosomal protein S12	x		
Traes_5BL_8601EC2E0	40S ribosomal protein S15a		x	
Traes_6BL_EFAF71592	40S ribosomal protein S24		x	
Traes_7AL_E7E665596	40S ribosomal protein S24		x	
Traes_7BL_0F1408688	40S ribosomal protein S24	x		
Traes_5AL_106BEC5D4	40S ribosomal protein S26			x
Traes_5BL_EC6277E0E	40S ribosomal protein S26			x
Traes_4AS_8ED92AF77	40S ribosomal protein S3a			x
Traes_4BL_7D2DDCA26	40S ribosomal protein S3a			x
Traes_4BL_66E76E6B0	40S ribosomal protein S3a	x		
Traes_1AS_E58B0B23A	40S ribosomal protein S4		x	
Traes_1BS_DD36A7A9B	40S ribosomal protein S4		x	
Traes_2AS_9CFA03D22	40S ribosomal protein S6			x
Traes_2AS_E08BF60FE	40S ribosomal protein S6			x
Traes_2BS_C41E04839	40S ribosomal protein S6			x
Traes_2AS_C7813CD47	40S ribosomal protein S6			x
Traes_2AL_1B2CC9926	40S ribosomal protein S8	x		
Traes_2BL_7BC3942D6	40S ribosomal protein S8	x		
Traes_2AS_3F458D2CF	40S ribosomal protein SA	x		
Traes_2BS_ECC74F149	40S ribosomal protein SA	x		
Traes_3AS_7F20E18E2	50S ribosomal protein 5, chloroplastic	x		
Traes_1BL_8AC8D124C	50S ribosomal protein L1, chloroplastic			x
Traes_1AL_3B838C090	50S ribosomal protein L1, chloroplastic	x		
Traes_1AL_B01B26670	50S ribosomal protein L1, chloroplastic	x		
Traes_5BL_AA5911949	50S ribosomal protein L17, chloroplastic	x		
Traes_5BL_AA5911949	50S ribosomal protein L17, chloroplastic	x		

Traes_6AS_EB8BA8FE2	50S ribosomal protein L21, chloroplastic	x		
Traes_7AL_EA6B88D3F	50S ribosomal protein L24, chloroplastic	x		
Traes_7BL_C99BF5CA9	50S ribosomal protein L24, chloroplastic	x		
Traes_3AL_C134C28BC	50S ribosomal protein L27, chloroplastic			x
Traes_1BS_71C6FDF1A	50S ribosomal protein L28, chloroplastic	x		
Traes_6BL_A66A6DCB2	50S ribosomal protein L29, chloroplastic			x
Traes_6AL_33DE178C5	50S ribosomal protein L29, chloroplastic			x
Traes_6AS_478618F75	50S ribosomal protein L3-1, chloroplastic	x		
Traes_6BS_AEB9C88B3	50S ribosomal protein L3-1, chloroplastic	x		
Traes_3AS_DF92A1FC5	50S ribosomal protein L3-2, chloroplastic		x	
Traes_3AL_4EC74A033	50S ribosomal protein L34, chloroplastic	x		
Traes_7BL_7A7BC0D15	50S ribosomal protein L35	x		
Traes_4BS_6EAD366CD	50S ribosomal protein L6, chloroplastic	x		
Traes_4BS_6EAD366CD	50S ribosomal protein L6, chloroplastic	x		
Traes_4AS_3CD9834D6	50S ribosomal protein L6, chloroplastic			x
Traes_4AS_49782B510	50S ribosomal protein L6, chloroplastic			x
Traes_5AL_4E0638B3E	50S ribosomal protein L6, chloroplastic			x
Traes_7BL_86BB13ED9	60S ribosomal protein L13	x		
Traes_7AL_4A3378136	60S ribosomal protein L13	x		
Traes_3AL_7BCADDE08	60S ribosomal protein L18a		x	
Traes_5AL_0C11FAA4D	60S ribosomal protein L21	x		
Traes_3AL_350DFFE84	60S ribosomal protein L36	x		
Traes_5BS_26A43A8A0	60S ribosomal protein L36	x		
Traes_5AS_6D0CC3B1F	60S ribosomal protein L36			x
Traes_1BL_0FC423766	60S ribosomal protein L36			x
Traes_3AL_8131EBAD8	60S ribosomal protein L37a, expressed		x	
Traes_6BL_168034B0D	60S ribosomal protein L6	x		
Traes_6AL_88DE049AB	ACT domain-containing protein ACR3		x	
Traes_4BL_5AF52F028	Actin-depolymerizing factor 5			x
Traes_4AS_811353465	Actin-depolymerizing factor 5	x		
Traes_7AS_545BDB6F1	Alanine--tRNA ligase, chloroplastic/mitochondrial			x
Traes_5BL_E123D8905	ALBINO3-like protein 1, chloroplastic			x

Traes_6AL_CFE60316E	Annexin	x		
Traes_6AL_23B31796F	Aquaporin 7			x
Traes_6BL_0C17AE0D2	Armadillo repeat-containing protein LFR			x
Traes_2AS_0F4EA930D	Asparagine--tRNA ligase, chloroplastic/mitochondrial	x		
Traes_2AS_7D65E81D0	Asparagine--tRNA ligase, chloroplastic/mitochondrial			x
Traes_2BS_FF8887BE5	Asparagine--tRNA ligase, chloroplastic/mitochondrial			x
Traes_1AL_975CB04EA	ATP-dependent Clp protease proteolytic subunit			x
Traes_4AL_1F49A9D0D	ATP-dependent Clp protease proteolytic subunit	x		
Traes_7AS_090FDB9B1	ATP-dependent Clp protease proteolytic subunit	x		
Traes_4AS_C67AE6944	ATP-dependent Clp protease proteolytic subunit	x		
Traes_4BL_1D9E2AC36	ATP-dependent Clp protease proteolytic subunit	x		
Traes_1AL_3D9EA5952	ATP-dependent Clp protease proteolytic subunit-related protein 1, chloroplastic			x
Traes_7AL_356F6FF0E	Blue-light photoreceptor PHR2			x
Traes_3AL_12DCD0158	Cell division protein FtsY homolog, chloroplastic			x
Traes_2BL_6097FE67E	Cell division protein FtsZ homolog 1, chloroplastic	x		
Traes_2AL_086336B60	Chaperonin 60 subunit beta 4, chloroplastic	x		
Traes_2BL_5A94F1657	Chaperonin 60 subunit beta 4, chloroplastic	x		
Traes_2AL_64E61AB1B	CRM-domain containing factor CFM2, chloroplastic			x
Traes_2AL_49BBA1645	CRM-domain containing factor CFM2, chloroplastic	x		
Traes_2BL_8CCB9C88D	CRM-domain containing factor CFM2, chloroplastic	x		
Traes_6AL_86BABC721	Cryptochrome-2		x	
Traes_6AL_86BABC721	Cryptochrome-2		x	
Traes_5AL_EF0D9834F	Cysteine--tRNA ligase, chloroplastic/mitochondrial	x		
Traes_5BL_41F9FAA36	Cysteine--tRNA ligase, chloroplastic/mitochondrial	x		
Traes_5BL_0684B3A02	Cytochrome c biogenesis protein CCS1, chloroplastic	x		
Traes_2BS_642DC63EC	DEAD-box ATP-dependent RNA helicase 10			x
Traes_5AL_D952BA6DB	DEAD-box ATP-dependent RNA helicase 22			x
Traes_4AL_199EE1839	DEAD-box ATP-dependent RNA helicase 3			x
Traes_5BL_BD895CF84	DEAD-box ATP-dependent RNA helicase 3			x
Traes_4AL_199EE1839	DEAD-box ATP-dependent RNA helicase 3, chloroplastic	x		
Traes_5BL_BD895CF84	DEAD-box ATP-dependent RNA helicase 3, chloroplastic	x		

Traes_3AS_D097872BE	DEAD-box ATP-dependent RNA helicase 39	x		
Traes_3AS_F527FB55B	DEAD-box ATP-dependent RNA helicase 39	x		
Traes_6AL_D153AC732	DEAD-box ATP-dependent RNA helicase 47, mitochondrial	x		
Traes_6BL_3C4549AB1	DEAD-box ATP-dependent RNA helicase 47, mitochondrial	x		
Traes_5AL_B72E1DD70	DEAD-box ATP-dependent RNA helicase 50	x		
Traes_3B_F4870ACB3	DEAD-box ATP-dependent RNA helicase 50	x		
Traes_3B_420994D45	DEAD-box ATP-dependent RNA helicase 58, chloroplastic	x		
Traes_3AS_30D175BEB	DNA gyrase subunit B		x	
Traes_2AL_61B7762C7	DNA ligase 4			x
Traes_5BL_A60442138	DNA mismatch repair protein MSH6			x
Traes_3AL_E77A5F03A	DNA-directed RNA polymerase subunit			x
Traes_1BS_9F8A8021B	DNA-directed RNA polymerase subunit			x
Traes_6BS_54B2B1260	DNA-directed RNA polymerase subunit			x
Traes_6BS_C85FE9482	DNA-directed RNA polymerase V subunit 1			x
Traes_1AS_AEEC8D740	E3 ubiquitin-protein ligase UPL6			x
Traes_2AS_7D3EC9FC2	E3 ubiquitin-protein ligase		x	
Traes_5AL_87C8496BE	Elongation factor 1-alpha	x		
Traes_2AL_837BCD21C	Elongation factor G, chloroplastic			x
Traes_5AS_91BEB6A34	Elongation factor Ts, mitochondrial	x		
Traes_6AL_32FADD06A	Elongation factor Tu	x		
Traes_6BL_A1759ED17	Elongation factor Tu	x		
Traes_6AL_54260E477	Epimerase family protein SDR39U1 homolog, chloroplastic			x
Traes_2AL_95D918793	Eukaryotic translation initiation factor 3 subunit H	x		
Traes_2BS_4A4C9BD80	Eukaryotic translation initiation factor 3 subunit M		x	
Traes_5AS_2C7433827	Eukaryotic translation initiation factor 5A	x		
Traes_5BS_160C44A80	Eukaryotic translation initiation factor 5A	x		
Traes_5BS_8FB0A0D95	Eukaryotic translation initiation factor 5A	x		
Traes_2AL_2DCC96CD9	Eukaryotic translation initiation factor 5A			x
Traes_4AL_E4470B79C	Flap endonuclease 1			x
Traes_5BL_213C8B59D	Formin-like protein		x	
Traes_3AS_CD8EF3E96	FRIGIDA-like protein		x	
Traes_7AL_492C9C1E4	Glutamate receptor (GLR)	x		

Traes_1BL_EC3E899FE	Glutamyl-tRNA reductase			x
Traes_2AL_4D97AE321	Glutamyl-tRNA(Gln) amidotransferase subunit A, chloroplastic/mitochondrial	x		
Traes_2BL_0118B58B3	Glutamyl-tRNA(Gln) amidotransferase subunit A, chloroplastic/mitochondrial			x
Traes_2BL_EEE4AF800	Glutamyl-tRNA(Gln) amidotransferase subunit A, chloroplastic/mitochondrial			x
Traes_2AL_783420E8B	Glutamyl-tRNA(Gln) amidotransferase subunit B, chloroplastic/mitochondrial	x		
Traes_6BS_C73BCAF43	Glutamyl-tRNA(Gln) amidotransferase subunit B, chloroplastic/mitochondrial	x		
Traes_4BL_9BA5AB82C	Glutamyl-tRNA(Gln) amidotransferase subunit B, chloroplastic/mitochondrial	x		
Traes_5AL_F55E09494	Glutamyl-tRNA(Gln) amidotransferase subunit C, chloroplastic/mitochondrial			x
Traes_6AL_96665E11C	GrpE protein homolog	x		
Traes_6BL_2CD26F5E7	GrpE protein homolog	x		
Traes_2BL_F3EE344D7	GrpE protein homolog	x		
Traes_2AL_18913037F	GrpE protein homolog			x
Traes_7BL_91C7CD5C5	GTP-binding nuclear protein		x	
Traes_4AS_F1F72C66C	GTP-binding protein At2g22870	x		
Traes_1BL_0A996D4A2	Histone H2B			x
Traes_2BL_BB2C0F695	Histone H3			x
Traes_5BL_71F88DB88	Histone H4			x
Traes_7BS_955956707	Histone-lysine N-methyltransferase CLF			x
Traes_2BL_392793376	Katanin p80 WD40 repeat-containing subunit B1 homolog		x	
Traes_2AL_A3F599522	Kinesin-like protein			x
Traes_3AS_BCC969DFF	Leucine--tRNA ligase, chloroplastic/mitochondrial			x
Traes_5AS_FC3C5CA46	LIM domain-containing protein WLIM1		x	
Traes_4AL_603B6DC64	LR34			x
Traes_6AL_0CD92304D	Lysine--tRNA ligase	x		
Traes_6BL_2936220F5	Lysine--tRNA ligase	x		
Traes_5BL_1DE088A30	Lysine--tRNA ligase	x		
Traes_5BL_CFC7BB483	Lysine--tRNA ligase	x		
Traes_6AS_2B85F4104	Mediator of RNA polymerase II transcription subunit 18			x

Traes_4AS_372712125	Methionine--tRNA ligase, chloroplastic/mitochondrial	x		
Traes_4BL_B19528CE1	Methionine--tRNA ligase, chloroplastic/mitochondrial	x		
Traes_4AS_6CE64CB5B	Microtubule-associated protein 70-5		x	
Traes_4BL_1C570A289	Microtubule-associated protein 70-5			x
Traes_7AL_D7CCFE55C	Multiple organellar RNA editing factor 9, chloroplastic	x		
Traes_7BL_FD98DF9E5	Multiple organellar RNA editing factor 9, chloroplastic	x		
Traes_1BL_BAA0AE22B	Nascent polypeptide-associated complex subunit beta	x		
Traes_4BL_70F08F293	Nascent polypeptide-associated complex subunit beta	x		
Traes_5AL_AB1566CF9	Nascent polypeptide-associated complex subunit beta	x		
Traes_1AL_61DFD90BF	Nascent polypeptide-associated complex subunit beta			x
Traes_7AL_48612DFF5	Novel plant SNARE 11		x	
Traes_4AL_4A747B462	Nuclear poly(A) polymerase 1			x
Traes_5BL_A9BFB8F58	Pectin acetylesterase	x		
Traes_5AL_85C8D32D0	Pectin acetylesterase	x		
Traes_3AL_0A528281C	Pectin acetylesterase	x		
Traes_1BL_698AC1335	Pentatricopeptide repeat-containing protein At1g15510, chloroplastic			x
Traes_2BL_B4DCEEB99	Pentatricopeptide repeat-containing protein At2g41720			x
Traes_4BS_8D508569E	Pentatricopeptide repeat-containing protein At3g06430, chloroplastic			x
Traes_7AS_CA6519CDB	Pentatricopeptide repeat-containing protein At3g18110, chloroplastic		x	
Traes_2AL_09781134C	Pentatricopeptide repeat-containing protein At3g22150, chloroplastic			x
Traes_2BL_EB51A1B4A	Pentatricopeptide repeat-containing protein At3g22150, chloroplastic			x
Traes_5AL_B84365C59	Pentatricopeptide repeat-containing protein At3g22150, chloroplastic			x
Traes_4AL_17C4B47B3	Pentatricopeptide repeat-containing protein At4g31850, chloroplastic		x	
Traes_5AL_64A3B1812	Pentatricopeptide repeat-containing protein At4g39620, chloroplastic	x		
Traes_6BL_34B508140	Pentatricopeptide repeat-containing protein At4g39620, chloroplastic	x		
Traes_2AL_9B7FE4EC3	Pentatricopeptide repeat-containing protein At5g50280, chloroplastic			x

Traes_2BL_18A640F3B	Pentatricopeptide repeat-containing protein At5g50280, chloroplastic			x
Traes_4BL_097C26EB0	Peptide chain release factor APG3, chloroplastic			x
Traes_1BS_F7B1A6268	Peptidyl-prolyl cis-trans isomerase	x		
Traes_3AS_12D74A531	Peptidyl-prolyl cis-trans isomerase CYP20-3, chloroplastic			x
Traes_6BS_387A98C03	Peptidyl-prolyl cis-trans isomerase FKBP18, chloroplastic			x
Traes_5AL_F8FD20EF8	Polyadenylate-binding protein	x		
Traes_2BS_23B5153F9	Polyribonucleotide nucleotidyltransferase 1, chloroplastic			x
Traes_2BS_F66779538	Polyribonucleotide nucleotidyltransferase 1, chloroplastic	x		
Traes_7AL_569D0BB1B	Pre-mRNA cleavage factor Im 25 kDa subunit 1	x		
Traes_1BL_A127DF6A9	Probable transmembrane GTPase FZO-like, chloroplastic			x
Traes_1AL_4BF6C25E1	Probable transmembrane GTPase FZO-like, chloroplastic			x
Traes_1BL_241A3B9EF	Probable ubiquitin-conjugating enzyme E2 24			x
Traes_5BL_196F297B6	Probable ubiquitin-conjugating enzyme E2 24			x
Traes_3AS_4C0261CD5	Probable zinc metalloprotease EGY2, chloroplastic			x
Traes_3AS_922982272	Probable zinc metalloprotease EGY2, chloroplastic	x		
Traes_5BL_5F8443EFD	Protein ACCUMULATION AND REPLICATION OF CHLOROPLASTS 3			x
Traes_1BS_CE144DF81	Protein ACCUMULATION AND REPLICATION OF CHLOROPLASTS 6, chloroplastic	x		
Traes_6AS_62C8C07B6	Protein ACCUMULATION AND REPLICATION OF CHLOROPLASTS 6, chloroplastic	x		
Traes_6AS_87906149C	Protein CHLORORESPIRATORY REDUCTION 6, chloroplastic			x
Traes_6BS_C7BF687C2	Protein CHLORORESPIRATORY REDUCTION 6, chloroplastic			x
Traes_6BS_63FCF7A14	Protein CHLORORESPIRATORY REDUCTION 6, chloroplastic		x	
Traes_2BL_985C16D8A	Proteinaceous RNase P 1, chloroplastic/mitochondrial			x
Traes_2AL_D31914DE6	Proteinaceous RNase P 1, chloroplastic/mitochondrial			x
Traes_2AL_D31914DE6	Proteinaceous RNase P 1, chloroplastic/mitochondrial	x		
Traes_4AL_345A5A89F	Putative 30S ribosomal protein S13			x
Traes_5AL_EDE003529	Putative ribosomal protein S18		x	
Traes_3AL_31E49AA0F	Putative translation initiation factor			x
Traes_7BL_8F226AC0C	Reticulon-like protein	x		

Traes_2AL_366D94C0B	Reticulon-like protein	x		
Traes_7AS_CDF144498	Rhomboid-like protein		x	
Traes_7AS_235A8B4D8	Ribonuclease E/G-like protein, chloroplastic		x	
Traes_7AS_97BCB0125	Ribonuclease E/G-like protein, chloroplastic		x	
Traes_5AL_CFEF57F4A	Ribonuclease Z, chloroplastic	x		
Traes_5BL_19748672B	Ribonuclease Z, chloroplastic	x		
Traes_3AL_137E874E4	Ribosomal protein	x		
Traes_3AL_74778D9DF	Ribosomal protein	x		
Traes_2AS_76163A005	Ribosomal protein L15	x		
Traes_2BS_100054707	Ribosomal protein L15		x	
Traes_1BL_C706FAA2B	Ribosomal protein L17		x	
Traes_6AL_288540CF3	Ribosomal protein L17	x		
Traes_1AL_D20D648FD	Ribosomal protein L17			x
Traes_6BL_30EFB4704	Ribosomal protein L17			x
Traes_2AS_C750EF91E	Ribosomal protein L19		x	
Traes_2BS_A9BF58434	Ribosomal protein L19		x	
Traes_2BS_CB8DF8A02	Ribosomal protein L19		x	
Traes_2AS_E1D0B2CE3	Ribosomal protein L19		x	
Traes_2AS_467FD5266	Ribosomal protein L19			x
Traes_2BS_7135D68C5	Ribosomal protein L19			x
Traes_6AL_D5111DA34	Ribosomal protein L37	x		
Traes_5AS_8785C0B49	Ribosomal protein L3-A3	x		
Traes_7AL_A1EA63AD2	Ribosomal protein P1			x
Traes_4AL_001CD0D65	Ribosomal protein S20		x	
Traes_2AL_16CF7A3BA	Ribosomal protein S20	x		
Traes_4AS_98267C6F6	Ribosomal protein S7	x		
Traes_4BL_BF17A941B	Ribosomal protein S7	x		
Traes_2AL_EC2CE85AC	RING-type E3 ubiquitin transferase			x
Traes_2AL_F5A6D2E35	RNA-binding protein CP33, chloroplastic	x		
Traes_2BL_8CC7DE748	RNA-binding protein CP33, chloroplastic	x		
Traes_3AS_ECAACF24F	RNA-dependent RNA polymerase	x		
Traes_3B_2C6DB84FB	RNA-dependent RNA polymerase	x		

	Traes_6AL_13BC97E04	RNA-dependent RNA polymerase			x
	Traes_4AL_5D769AED8	RuvB-like helicase			x
	Traes_5BS_5361C483D	Synonyms	x		
	Traes_2BL_FE46FC938	TaAP2-B	x		
	Traes_7AS_378A12AA9	TGW-7A		x	
	Traes_5BL_369BF7271	Thioredoxin-like protein HCF164, chloroplastic			x
	Traes_1BL_F1640B356	Thylakoid lumenal 17.4 kDa protein, chloroplastic			x
	Traes_1AL_5558B7A4C	Thylakoid lumenal 17.4 kDa protein, chloroplastic	x		
	Traes_1AL_5558B7A4C	Thylakoid lumenal 17.4 kDa protein, chloroplastic	x		
	Traes_4AS_553C18F16	Translocase of chloroplast	x		
	Traes_4BL_E48C704F1	Translocase of chloroplast	x		
	Traes_5AL_A9C9D731A	Triticain gamma			x
	Traes_2BS_E5A5144E0	tRNA (guanine(26)-N(2))-dimethyltransferase			x
	Traes_7AL_6ED296411	tRNA dimethylallyltransferase 9		x	
	Traes_3AL_7494F4783	Tryptophan--tRNA ligase, chloroplastic/mitochondrial			x
	Traes_1AL_FE1097B22	Tyrosine--tRNA ligase, chloroplastic/mitochondrial	x		
	Traes_6AS_E011BC5BB	Ubiquitin-conjugating enzyme E2 29		x	
	Traes_2AL_496A24411	Ubiquitin-like-conjugating enzyme ATG10	x		
	Traes_4BL_5FCA04C3B	YlmG homolog protein 2, chloroplastic			x
