# Supplementary material for: Comparative Analysis Based on Transcriptomics and Metabolomics Data Reveal Differences between Emmer and Durum Wheat in Response to Nitrogen Starvation
Source: Int J Mol Sci. 2021 Apr 30;22(9):4790. doi: 10.3390/ijms22094790 (PMC8124848; doi:10.3390/ijms22094790)
Supplement: Supplementary file 1 [file ijms-22-04790-s001.zip › Suppl.Table S3.pdf]

**Table S3.** Number of DEGs (up-/down-regulated) under two N conditions, enriched GO terms before and after FDR correction in emmer (Molise Sel. Colli), durum wheat (Simeto), and in total.

| Genotypes | up/ down | DE genes | GO enriched | Filtered GO (FDR) |
|---|---|---|---|---|
| emmer (Molise Sel. Colli) | up | 1202 | 486 | 390 |
| | down | 586 | 116 | 94 |
| durum wheat (Simeto) | up | 1846 | 621 | 477 |
| | down | 1283 | 239 | 178 |
| total | up | 3048 | 1107 | 867 |
| | down | 1869 | 355 | 272 |
