# Supplementary material for: Comparative Analysis Based on Transcriptomics and Metabolomics Data Reveal Differences between Emmer and Durum Wheat in Response to Nitrogen Starvation
Source: Int J Mol Sci. 2021 Apr 30;22(9):4790. doi: 10.3390/ijms22094790 (PMC8124848; doi:10.3390/ijms22094790)
Supplement: Supplementary file 1 [file ijms-22-04790-s001.zip › Suppl.Table S4.pdf]

Table S4. Most significant GO categories in emmer (Molise Sel. Colli) and durum wheat (Simeto)

	categories	GO Terms	p_value emmer	Number of DEGs per GO emmer	p_value durum wheat	Number of DEGs per GO durum wheat
Biological Process up-regulated	cellular component organization or biogenesis	plastid organization			1,21E-14	67
		chloroplast organization			3,16E-10	50
	cellular process	translation	1,40E-47	181	1,12E-82	273
		peptide biosynthetic process	2,50E-46	181	1,61E-81	274
		amide biosynthetic process	1,08E-45	185	2,08E-82	283
		peptide metabolic process	1,81E-45	182	1,80E-79	275
		cellular amide metabolic process	8,71E-43	186	2,98E-77	284
		cellular nitrogen compound biosynthetic process	8,64E-18	283	1,25E-29	417
		cellular biosynthetic process	4,41E-16	366	4,15E-25	535
		cellular nitrogen compound metabolic process	6,07E-11	367	3,48E-19	540
		carboxylic acid metabolic process	1.45E-05	148	2,21E-07	203
		plastid organization			1,21E-14	67
		chloroplast organization			3,16E-10	50
		ncRNA metabolic process			2,05E-08	102
		cellular amino acid metabolic process			2,34E-08	134
		oxoacid metabolic process			1,24E-06	204
		organic acid metabolic process			1,61E-06	204
		tetrapyrrole biosynthetic process			5,50E-06	39
		isopentenyl diphosphate metabolic process			9,20E-06	40
		isopentenyl diphosphate biosynthetic process			9,20E-06	40
		cellular macromolecule biosynthetic process			1,49E-05	349
		isopentenyl diphosphate biosynthetic process, methylerythritol 4-phosphate pathway			1,55E-05	39
	metabolic process	organonitrogen compound biosynthetic process	2,07E-68	286	2,82E-100	412
		organonitrogen compound metabolic process	3,33E-49	310	2,21E-78	459
		translation	1,40E-47	181	1,12E-82	273
		peptide biosynthetic process	2,50E-46	181	1,61E-81	274
		amide biosynthetic process	1,08E-45	185	2,08E-82	283

		peptide metabolic process	1,81E-45	182	1,80E-79	275
		cellular amide metabolic process	8,71E-43	186	2,98E-77	284
		cellular nitrogen compound biosynthetic process	8,64E-18	283	1,25E-29	417
		organic substance biosynthetic process	8,30E-17	372	1,24E-21	532
		cellular biosynthetic process	1,41E-16	366	4,15E-25	535
		biosynthetic process	2,21E-14	381	2,72E-20	552
		nitrogen compound metabolic process	4,72E-13	399	6,40E-22	591
		cellular nitrogen compound metabolic process	6,07E-11	367	3,48E-19	540
		carboxylic acid metabolic process	1,45E-05	148	2,21E-07	203
		gene expression			2,00E-11	385
		ncRNA metabolic process			2,05E-08	102
		cellular amino acid metabolic process			2,34E-08	134
		oxoacid metabolic process			1,24E-06	204
		organic acid metabolic process			1,61E-06	204
		tetrapyrrole biosynthetic process			5,50E-06	39
		isopentenyl diphosphate biosynthetic process			9,20E-06	40
		isopentenyl diphosphate metabolic process			9,20E-06	40
		cellular macromolecule biosynthetic process			1,49E-05	349
		isopentenyl diphosphate biosynthetic process, methylerythritol 4-phosphate pathway			1,55E-05	39
		macromolecule biosynthetic process			6,97E-05	351
Molecular Function up-regulated	binding	binding	1,68E-39	507	0	736
		RNA binding	2,22E-19	92	7,35E-44	127
		heterocyclic compound binding	4,44E-17	330	5,31E-80	469
		organic cyclic compound binding	4,44E-17	330	5,31E-80	469
		nucleic acid binding	4,19E-07	163	1,19E-29	222
		ion binding			9,23E-46	404
		small molecule binding			8,90E-21	279
		nucleoside phosphate binding			3,66E-16	267
		nucleotide binding			3,66E-16	267
		anion binding			2,81E-14	249
		purine ribonucleoside triphosphate binding			6,14E-05	196
	catalytic activity	catalytic activity	4,90E-94	518	0	740

		hydrolase activity	5,99E-09	182	1,27E-26	234
		oxidoreductase activity			1,27E-26	159
		ligase activity			5,35E-16	75
		ligase activity, forming aminoacyl-tRNA and related compounds			6,69E-14	41
		ligase activity, forming carbon-oxygen bonds			6,69E-14	41
		hydrolase activity, acting on glycosyl bonds			1,47E-13	64
		aminoacyl-tRNA ligase activity			3,00E-13	41
		hydrolase activity, hydrolyzing O-glycosyl compounds			8,83E-12	60
		lyase activity			6,98E-10	57
		transferase activity, transferring one-carbon groups			7,72E-05	51
	structural molecule activity	structural constituent of ribosome	1,16E-92	141	1,12E-160	196
		structural molecule activity	7,74E-84	146	6,84E-141	198
Molecular Function down-regulated	binding	binding			0	556
		heterocyclic compound binding			3,70E-90	390
		organic cyclic compound binding			3,70E-90	390
		ion binding			1,92E-74	363
		anion binding			9,34E-50	261
		adenyl ribonucleotide binding			5,67E-45	226
		adenyl nucleotide binding			6,05E-45	228
		carbohydrate derivative binding			5,22E-42	238
		ribonucleotide binding			4,16E-41	234
		ATP binding			1,05E-40	209
		ribonucleoside binding			1,41E-39	232
		nucleoside binding			1,45E-39	232
		purine nucleoside binding			2,18E-39	231
		purine ribonucleoside binding			2,18E-39	231
		purine ribonucleotide binding			2,18E-39	231
		purine nucleotide binding			2,47E-39	233
		nucleoside phosphate binding			7,26E-37	252
		nucleotide binding			7,26E-37	252
		small molecule binding			2,61E-36	254
		purine ribonucleoside triphosphate binding			5,09E-35	214

catalytic activity	catalytic activity			0	566
	transferase activity			1,23E-36	228
	transferase activity, transferring phosphorus-containing groups			2,45E-28	160
	kinase activity			3,96E-25	140
	phosphotransferase activity, alcohol group as acceptor			1,49E-22	134
	protein kinase activity			1,98E-21	122
	hydrolase activity			2,00E-16	174
	protein serine/threonine kinase activity			1,06E-05	58
transporter activity	transporter activity			3,12E-36	120
	transmembrane transporter activity			1,93E-19	84
	substrate-specific transporter activity			2,79E-06	60
	active transmembrane transporter activity			9,09E-05	42
