# Supplementary material for: Comparative Analysis Based on Transcriptomics and Metabolomics Data Reveal Differences between Emmer and Durum Wheat in Response to Nitrogen Starvation
Source: Int J Mol Sci. 2021 Apr 30;22(9):4790. doi: 10.3390/ijms22094790 (PMC8124848; doi:10.3390/ijms22094790)
Supplement: Supplementary file 1 [file ijms-22-04790-s001.zip › Suppl.Table S5.pdf]

Table S5. ANOVA for each individual metabolite, relating to their content in the leaves of emmer and durum wheat grown under starvation and optimal N conditions.

	**G**	**N**	**G x N**
valine	ns	ns	***
glutamic acid	***	***	***
Alanine	ns	ns	***
β-alanine	ns	ns	***
Serine	ns	ns	**
glycine	ns	ns	ns
Threonine	ns	ns	*
aspartic acid	**	*	***
asparagine	ns	ns	ns
thryptophan	*	ns	ns
GABA	ns	ns	**
citric acid	**	**	**
aconitic acid	**	ns	***
isocitric acid	ns	ns	ns
succinic acid	ns	ns	*
fumaric acid	ns	ns	**
malic acid	ns	ns	**
shikimic acid	*	ns	**
quinic acid	ns	ns	***
gluconic acid	ns	ns	ns
saccharic acid	**	*	**
oxalic acid	ns	ns	ns
phosphate	ns	ns	ns
xylose - lyxose - threalose	ns	ns	ns
ribose	ns	ns	**
Fructose	ns	ns	ns
mannose - galactose - glucose	ns	ns	ns
sucrose	ns	ns	ns
isomaltose	ns	ns	*
maltose - turanose	ns	ns	**
lactulose	ns	ns	ns
maltitol	***	***	***
glycerol	ns	ns	ns
mannitol	ns	ns	ns
sorbitol	ns	ns	ns
myo-inositol	ns	ns	**
hexadecanoic acid	ns	ns	ns
1-octacosanol	ns	ns	ns
campsterol	ns	ns	ns
stigmasterol	ns	ns	ns
β-sitosterol	ns	ns	ns

n.s., not significant; *, $P \leq 0.05$, **, $P \leq 0.01$; ***, $P \leq 0.001$
