# Supplementary material for: Comparative Analysis Based on Transcriptomics and Metabolomics Data Reveal Differences between Emmer and Durum Wheat in Response to Nitrogen Starvation
Source: Int J Mol Sci. 2021 Apr 30;22(9):4790. doi: 10.3390/ijms22094790 (PMC8124848; doi:10.3390/ijms22094790)
Supplement: Supplementary file 1 [file ijms-22-04790-s001.zip › Suppl.Table S6.pdf]

Table S6. Number of edges between central DEGs genes and significantly behaved metabolites under the two N conditions. For each metabolite was highlight in bold the higher number of edges.

From	To	wheat	Total edge between DEGs and significantly behaved metabolites	Total edge between central DEGs and significantly behaved metabolites
DEGs	Aconitic acid	emmer<br>durum wheat	383<br>1867	71<br>**344**
DEGs	Alanine	emmer<br>durum wheat	705<br>1430	88<br>**320**
DEGs	aspartic acid	emmer<br>durum wheat	899<br>497	143<br>119
DEGs	β-alanine	emmer<br>durum wheat	37<br>687	10<br>**179**
DEGs	citric acid	emmer<br>durum wheat	700<br>464	130<br>124
DEGs	fumaric acid	emmer<br>durum wheat	527<br>548	89<br>121
DEGs	GABA	emmer<br>durum wheat	12<br>2954	1<br>**479**
DEGs	glutamic acid	emmer<br>durum wheat	1360<br>101	**201**<br>19
DEGs	isocitric acid	emmer<br>durum wheat	44<br>53	**21**<br>7
DEGs	Isomaltose	emmer<br>durum wheat	338<br>28	**55**<br>0
DEGs	malic acid	emmer<br>durum wheat	213<br>851	54<br>**199**
DEGs	maltose-turanose	emmer<br>durum wheat	254<br>1796	36<br>**299**
DEGs	myo-inositol	emmer<br>durum wheat	36<br>2725	5<br>**460**
DEGs	maltitol	emmer<br>durum wheat	1667<br>1977	431<br>391
DEGs	quinic acid	emmer<br>durum wheat	244<br>2873	60<br>**474**
DEGs	Ribose	emmer<br>durum wheat	131<br>1906	36<br>**355**
DEGs	saccharic acid	emmer<br>durum wheat	52<br>59	**23**<br>2
DEGs	Serine	emmer<br>durum wheat	486<br>36	**77**<br>1
DEGs	shikimic acid	emmer<br>durum wheat	244<br>1564	52<br>**270**
DEGs	succinic acid	emmer<br>durum wheat	218<br>52	**47**<br>0
DEGs	Threonine	emmer<br>durum wheat	266<br>50	**50**<br>0
DEGs	Valine	emmer<br>durum wheat	1390<br>2484	218<br>**427**
