# Supplementary material for: Comparative Analysis Based on Transcriptomics and Metabolomics Data Reveal Differences between Emmer and Durum Wheat in Response to Nitrogen Starvation
Source: Int J Mol Sci. 2021 Apr 30;22(9):4790. doi: 10.3390/ijms22094790 (PMC8124848; doi:10.3390/ijms22094790)
Supplement: Supplementary file 1 [file ijms-22-04790-s001.zip › Suppl.Table S7.pdf]

Table S7. Putative functions of common DEGs in both tetraploid wheats with central roles in either of emmer-specific and durum wheat-specific networks.

DEGs	up/down	metabolites	group	Putative function description	Orthologues	Central role emmer	Central role durum wheat
Traes_1BL_AAC4D956E	*down*	Aconitic acid, aspartic acid, citric acid, fumaric acid, glutamic acid, maltitol, valine	C metabolism (glycolysis)	Pyruvate, phosphate dikinase 1(PPDK) (EC 2.7.9.1)	AT4G15530	**x**	
Traes_2AL_F1C4FF20C	*down*	Citric acid, glutamic acid, maltitol, valine	Fatty acid metabolism	Lecithin-cholesterol acyltransferase-like 4 (EC 2.3.1-)	AT4G19860	**x**	
Traes_1AL_053F6B12B	*up*	Alanine, aspartic acid, b-alanine, fumaric acid, glutamic acid, isocitric acid, isomaltose, malic acid, maltose-turanose, maltitol, quinic acid, ribose, saccharic acid, serine, shikimic acid, succinic acid, threonine, valine	C metabolism (photosynthesis)	Chlorophyll synthase (CHLG) (EC 2.5.1.62)	AT3G51820	**x**	
Traes_1AL_FE1097B22	*up*	Maltitol	Others	Tyrosine--tRNA ligase, chloroplastic/mitochondrial (EC 6.1.1.1)	AT3G02660	**x**	
Traes_2AS_F485758C1	*up*	Citric acid, glutamic acid, maltitol, valine	C metabolism (glycolysis)	Pyruvatedehydrogenase E1 component subunit alpha-3 (PDH-E1 ALPHA) (EC 1.2.4.1)	AT1G01090	**x**	
Traes_2BS_959A4E58A	*up*	Citric acid, glutamic acid, maltitol, valine	Kinases	Fructokinase-like 2 (FLN 2)	AT1G69200	**x**	
Traes_4AL_45E27280D	*up*	Alanine, serine, valine, maltitol	Amino acid metabolism	Cysteine desulfurase 1 (NIFS) (EC 2.8.1.7)	AT1G08490	**x**	
Traes_3AS_D097872BE	*up*	Aconitic acid, alanine, aspartic acid, fumaric acid, glutamic acid, malic acid, maltitol, valine	Transcription factors	DEAD-box ATP-dependent RNA helicase 39 (EC 3.6.4.13)	AT4G09730	**x**	
Traes_6AS_FD8F6B539	*up*	Maltitol, valine	Stress	Rhodanese-like domain-containing protein 7 (STR 7)	AT2G40760	**x**	
Traes_7AL_A1ECABC74	*up*	Alanine, fumaric acid, glutamic acid, isocitric acid, isomaltose, maltitol, saccharic acid, serine, threonine	Others	30S ribosomal protein S31	AT2G38140	**x**	
Traes_7BS_3285CDC24	*up*	Fumaric acid, threonine	Stress	F-box protein MAX2	AT2G42620	**x**	

Traes_2BL_CCD296233	*down*	Emmer (Citric acid, glutamic acid, maltitol, valine)<br><br>Durum wheat (Aconitic acid, alanine, GABA, malic acid, maltose-turanose, myo-inositol, maltitol, quinic acid, ribose, valine)	Stress	Stress enhanced protein 2 (SEP2)	AT2G21970	**x**	**x**
Traes_2AL_DB64E18A1	*down*	GABA, myo-inositol, maltitol, quinic acid, ribose, valine	Purine metabolism	Allantoinase (ALN) (EC 3.5.2.5)	AT4G04955		**x**
Traes_7BS_7E9C070B8	*down*	Aconitic acid, alanine, b-alanine, citric acid, GABA, malic acid, maltose-turanose, myo-inositol, maltitol, quinic acid, shikimic acid, valine	C metabolism (photorespiration)	Carboxyl-terminal-processing peptidase 3 (CTPA3)<br><br>(EC 3.4.21.102)	AT3G57680		**x**
Traes_1AL_928DC3A3C	*up*	Aconitic acid, alanine, aspartic acid, citric acid, GABA, maltose-turanose, myo-inositol, maltitol, quinic acid, ribose, shikimic acid, valine	Riboflavine metabolism	Bifunctional riboflavin kinase/FMN phosphatase (FMN/FHY)<br>(EC 3.1.3.102/ EC 2.7.1.26)	AT4G21470		**x**
Traes_3AL_8C6F4F66A	*up*	Aconitic acid, citric acid, GABA, maltose-turanose, myo-inositol, quinic acid, ribose, shikimic acid, valine	Terpenoid backbone biosynthesis	4-diphosphocytidyl-2-C-methyl-D-erythritol kinase (ISPE)<br>(EC 2.7.1.148)	AT2G26930		**x**
Traes_3AS_922982272	*up*	Alanine, b-alanine, fumaric acid	C metabolism (glycolisis)	Probable zinc metalloprotease EGY2 (EC 3.4.24-)	AT5G05740		**x**
Traes_4AS_0B56CEBD5	*up*	Aconitic acid, GABA, maltose-turanose, myo-inositol, quinic acid, ribose, shikimic acid, valine	stress	ATP-dependent Clp protease proteolytic subunit-related protein 3 (CLPR3)	AT1G09130		**x**
Traes_4BS_0ECAB85C2	*up*	Aconitic acid, GABA, isocitric acid, maltose-turanose, myo-inositol, quinic acid, ribose, valine	others	30S ribosomal protein S13	AT5G14320		**x**
Traes_4AL_199EE1839<br><br>Traes_5BL_BD895CF84	*up*	Aconitic acid, alanine, citric acid, GABA, malic acid, maltose-turanose, myo-inositol, maltitol, quinic acid,	Transcription factors	DEAD-box ATP-dependent RNA helicase 3 (EC 3.6.4.13)	AT5G26742		x

		ribose, shikimic acid, valine<br><br>Aconitic acid, alanine, b-alanine, citric acid, GABA, malic acid, maltose-turanose, myo-inositol, maltitol, quinic acid, ribose, shikimic acid, valine					
Traes_5BL_0684B3A02	*up*	Aconitic acid, alanine, citric acid, GABA, maltose-turanose, myo-inositol, maltitol, quinic acid, ribose, shikimic acid, valine	Energy pathways	Cytochrome c biogenesis protein (CCS1)	AT1G49380		**x**
Traes_5BL_CFC7BB483	*up*	Aconitic acid, alanine, citric acid, GABA, maltose-turanose, myo-inositol, maltitol, quinic acid, ribose, shikimic acid, valine	Porphyrin and chlorophyll metabolism	Magnesium-chelatase subunit ChlD (EC 6.6.1.1)	AT1G08520		**x**
Traes_6BL_34B508140	*up*	Aconitic acid, alanine, GABA, malic acid, maltose-turanose, myo-inositol, maltitol, quinic acid, valine	others	Pentatricopeptide repeat-containing protein At4g39620	AT4G39620		**x**
