# Supplementary material for: Comparative Analysis Based on Transcriptomics and Metabolomics Data Reveal Differences between Emmer and Durum Wheat in Response to Nitrogen Starvation
Source: Int J Mol Sci. 2021 Apr 30;22(9):4790. doi: 10.3390/ijms22094790 (PMC8124848; doi:10.3390/ijms22094790)
Supplement: Supplementary file 1 [file ijms-22-04790-s001.zip › Suppl.Table S8.pdf]

Table S8. Putative function of DEGs specific to each of the tetraploid wheats with central roles in either of emmer-specific and durum wheat-specific networks.

DEGs	up/down	metabolites	group	Putative function description	Orthologues	Central role emmer	Central role durum wheat
Traes_1AL_5F0F4FAFB	*down*	aspartic acid, citric acid glutamic acid, maltitol valine	kinases	Non-specific serine/threonine protein kinase (EC 2.7.11.1)		**x**	
Traes_3AL_883A4805C	*down*	aconitic acid, aspartic acid, citric acid, fumaric acid, glutamic acid, maltitol, valine	other C metabolisms	Cytokinin riboside 5'-monophosphate phosphoribohydrolase (EC 3.2.2.n1)	AT2G37210	**x**	
Traes_4BL_4D370C9FA	*down*	Alanine, fumaric acid glutamic acid, maltitol, threonine	stress	Probable mediator of RNA polymerase II transcription subunit 37c	AT3G12580	x	
Traes_4BL_4D370C9FA1		Alanine, fumaric acid glutamic acid, isomaltose, maltitol serine, valine					
Traes_4BL_96C906D61	*down*	citric acid, glutamic acid maltitol, valine	stress	Non-specific lipid-transfer protein	AT3G08770	**x**	
Traes_5AL_6FB526190	*down*	citric acid, glutamic acid maltitol, valine	N metabolism	Carbonic anhydrase (EC 4.2.1.1)	AT5G14740	**x**	
Traes_5BL_A0ED5623C	*down*	Maltitol, valine	stress	Phospholipase D delta (PLDDELTA**)** (EC3.1.4.4)	AT4G35790	**x**	
Traes_7AS_9A01E6B17	*down*	aconitic acid, aspartic acid, citric acid, glutamic acid, maltitol valine	transporter	Potassium transporter	AT2G30070	**x**	
Traes_7BL_8930CD1E5	*down*	aspartic acid, citric acid glutamic acid, palatinose -maltitol, valine	Stress	Peptide methionine sulfoxide reductase B1, chloroplastic (EC 1.8.4.12)	AT1G53670	**x**	
Traes_1AL_4B7636BC9	*up*	Valine, maltitol	Stress	Protein LOL1	AT1G32540	**x**	
Traes_1AL_090571678	*up*	Alanine, aspartic acid fumaric acid, glutamic	transporter	Secretory carrier-associated membrane protein (SCAMP)	AT1G61250	**x**	

		acid, isomaltose, malic acid, maltose-turanose, maltitol quinic acid, ribose, serine, shikimic acid, valine				
Traes_1BL_5F74C58ED		alanine, glutamic acid, maltose-turanose, maltitol				
Traes_1AL_1538AC680	*up*	alanine, citric acid, glutamic acid, maltose-turanose, maltitol, valine	Kinases	Nucleoside diphosphate kinase (EC 2.7.4.6)	AT4G23895	**x**
Traes_1BL_ACC9DDA86		citric acid, glutamic acid maltitol, valine				
Traes_1AL_8E8E7401B	*up*	alanine, maltose-turanose, maltitol, serine, valine	other C metabolisms	Beta-hexosaminidase (EC 3.2.1.52)	AT3G49850	**x**
Traes_1BL_7F9006C51	*up*	glutamic acid, maltitol, valine	Stress	Hyperosmolality-gated Ca2+ permeable channel		**x**
Traes_1BL_C706FAA2B	*up*	glutamic acid maltitol, valine	Others	Ribosomal protein L17	AT3G54210	**x**
Traes_2AL_5BAB26827	*up*	citric acid, maltitol, valine	Stress	Lipoxygenase (EC 1.13.11-)	AT3G45140	**x**
Traes_2AS_34272605C	*up*	alanine, aspartic acid, fumaric acid, glutamic acid, isocitric acid isomaltose, maltitol, quinic acid, ribose, saccharic acid, serine shikimic acid, succinic acid, threonine, valine	Stress	Tubulin alpha chain	AT4G14960	**x**
Traes_2AS_7D3EC9FC2	*up*	citric acid, glutamic acid maltitol, valine	Others	E3 ubiquitin-protein ligase		**x**
Traes_3AS_CD8EF3E96	*up*	citric acid, glutamic acid maltitol, valine	Others	FRIGIDA-like protein	AT5G16320	**x**
Traes_4AL_17C4B47B3	*up*	aconitic acid, aspartic acid, glutamic acid,	Others	Pentatricopeptide repeat-containing protein At4g31850,	AT4G31850	**x**

		maltitol		chloroplastic			
Traes_4AS_4C25DF6F4	*up*	aspartic acid, citric acid glutamic acid, maltitol valine	others C metabolism	Beta-galactosidase (EC 3.2.1.23)	AT2G32810	**x**	
Traes_5AL_CEEDF43FC	*up*	alanine, aspartic acid, isomaltose, maltose-turanose, maltitol, ribose, serine, valine	Fatty acid metabolism	Acyl-[acyl-carrier-protein] hydrolase (EC 3.1.2-)	AXX17-AT1G08340	**x**	
Traes_6AL_006779D22	*up*	aconitic acid, alanine, aspartic acid, citric acid, fumaric acid, glutamic acid, malic acid, maltitol, quinic acid, serine, valine	other C metabolisms	Xyloglucan endotransglucosylase/hydrolase (EC 2.4.1.207)	AT2G06850	**x**	
Traes_6BS_C9B216D97	*up*	alanine, aspartic acid, citric acid, glutamic acid malic acid, maltitol quinic acid, valine	C metabolism (glycolysis)	Acetyltransferase component of pyruvate dehydrogenase complex (EC 2.3.1.12)	AXX17-AT1G48680	**x**	
Traes_7AS_378A12AA9	*up*	aconitic acid, aspartic acid, citric acid, fumaric acid, glutamic acid, maltitol, quinic acid, shikimic acid, valine	Others	TGW-7A		**x**	
Traes_7AS_A80BE362A	*up*	aconitic acid, aspartic acid, citric acid glutamic acid, maltitol valine	others C metabolism	Sucrose synthase (EC 2.4.1.13)	AT5G49190	**x**	
Traes_7BL_E2E7AA383	*up*	alanine, aspartic acid, fumaric acid, isocitric acid, isomaltose, myo-inositol, maltitol, quinic acid, ribose, serine, shikimic acid, succinic acid, threonine	Stress	Protein Overexpressor of Cationic Peroxidase 3	AT5G11270	**x**	
Traes_1AL_A970EBB27	*down*	aconitic acid, alanine,	Stress	Protein Detoxification 42	AT1G51340		**x**

		b-alanine, fumaric acid, GABA, malic acid, maltose-turanose, myo-inositol, maltitol, quinic acid, ribose, valine				
Traes_1AS_AEEC8D740	*down*	alanine, b-alanine, GABA, maltose-turanose, myo-inositol, maltitol, quinic acid, valine	Others	E3 ubiquitin-protein ligase UPL6 (EC 2.3.2.26)	AT3G17205	**x**
Traes_1BL_9772C6F45	*down*	alanine, aspartic acid beta-alanine, fumaric acid, GABA, malic acid myo-inositol, maltitol quinic acid, ribose, shikimic acid, valine	transporter	Calcium-transporting ATPase (EC 3.6.3.8)	AT1G27770	**x**
Traes_1BL_A9AC1340E	*down*	aconitic acid, alanine, GABA, malic acid, maltose-turanose, myo-inositol, maltitol quinic acid, ribose, valine	Transcription factors	Auxin response factor (ARF family)	AT5G20730	**x**
Traes_1BS_4A63D1DBC	*down*	GABA, quinic acid, ribose, shikimic acid, valine	other C metabolisms	Phosphatidate cytidylyltransferase (EC 2.7.7.41)	AT4G22340	**x**
Traes_2AL_BDC8DD6C7	*down*	aconitic acid, alanine aspartic acid, beta-alanine, fumaric acid, GABA, malic acid, maltose-turanose myo-inositol, maltitol, quinic acid, ribose, valine	kinases	Serine/threonine-protein kinase (EC 2.7.11.1)		**x**
Traes_2AL_E80950527	*down*	alanine, GABA, maltose-turanose, myo-inositol, maltitol, quinic acid, valine	transporter	Chloride channel protein	AT5G40890	**x**
Traes_2AL_EC2CE85AC	*down*	GABA, myo-inositol,	others	RING-type E3 ubiquitin	ATG23140	**x**

		quinic acid, ribose		transferase (EC 2.3.2.27)		
Traes_2AL_F4F6F518D	*down*	alanine, GABA, myo-inositol, quinic acid, ribose, maltitol	stress	Amine oxidase (EC 1.4.3-)	AT4G12290	**x**
Traes_2BL_19B3E60AA	*down*	aconitic acid, alanine, citric acid, GABA, malic acid, maltose-turanose, myo-inositol, maltitol, quinic acid, ribose, shikimic acid, valine	transporter	Probable copper-transporting ATPase HMA5 (EC 3.6.3.54)	AT1G63440	**x**
Traes_2BL_BB2C0F695	*down*	aconitic acid, alanine, beta-alanine, GABA myo-inositol, maltitol, quinic acid, ribose	others	Histone H3	AT5G65350	**x**
Traes_3AL_FFE4C92D8	*down*	aconitic acid, alanine, citric acid, GABA, malic acid, maltose-turanose, myo-inositol, maltitol, quinic acid, shikimic acid, valine	others C metabolism	Cellulose synthase A catalytic subunit 8 [UDP-forming] (EC 2.4.1.12)	AT4G18780	**x**
Traes_4AL_D7702A458	*down*	aconitic acid, beta-alanine, fumaric acid, GABA, myo-inositol, maltitol, quinic acid, ribose, shikimic acid, valine	transporter	Plasma membrane ATPase (EC 3.6.3.6)	AT2G18960	**x**
Traes_5AL_07C125B1C	*down*	alanine, beta-alanine, GABA, quinic acid, maltose-turanose, valine myo-inositol, shikimic acid, maltitol	stress	Hypersensitive induced reaction protein 2	AT1G69840	**x**
Traes_5AL_13E2DEC48	*down*	aconitic acid, GABA myo-inositol, valine, maltitol, quinic acid, ribose	Transcription factors	MIKC-type MADS-box transcription factor WM6		**x**
Traes_5AL_23B24917C	*down*	aconitic acid, alanine, beta-alanine, citric acid GABA, malic acid,	C metabolism (photosynthes is)	Ferrochelatase (EC 4.99.1.1)	AT2G30390	**x**

		maltose-turanose, valine				
		myo-inositol, shikimic acid, maltitol, quinic acid, ribose				
Traes_5BL_FF9AA4B58		aconitic acid, alanine, citric acid, GABA, maltose-turanose, myo-inositol, maltitol, quinic acid, ribose, shikimic acid, valine				
Traes_5AS_BA57B5B56	*down*	aconitic acid, GABA, myo-inositol, valine maltitol, quinic acid, ribose	Purine/pyrimidine metabolism	Ureidoglycolate hydrolase (allantoate amido hydrolase) (EC 3.5.1.116)	AT5G43600	**x**
Traes_5BL_369BF7271	*down*	GABA, myo-inositol, maltitol, quinic acid	others	Thioredoxin-like protein HCF164	AT4G37200	**x**
Traes_5BL_E123D8905	*down*	aconitic acid, alanine, citric acid, GABA, malic acid, maltose-turanose, myo-inositol, maltitol, quinic acid, ribose, valine	others	ALBINO3-like protein 1, chloroplastic	AT1G24490	**x**
Traes_6AS_0C1D497EA	*down*	aconitic acid, alanine, b-alanine, citric acid, GABA, malic acid, valine, maltose-turanose, myo-inositol, maltitol, quinic acid, ribose, shikimic acid	Amino acid metabolism	S-adenosylmethionine synthase 4 (EC 2.5.1.6)	AT3G17390	**x**
Traes_6AS_F3CC61A36	*down*	GABA, quinic acid, shikimic acid, valine	other C metabolisms	Terpene cyclase/mutase family member (EC5.4.99-)	AT1G78950	**x**
Traes_6BL_2B67C5E30	*down*	aconitic acid, GABA, maltose-turanose, myo-inositol, quinic acid, valine	transporter	Glycolipid transfer protein 1 (GLTP 1)	AT2G33470	**x**
Traes_6BS_A80520954	*down*	aconitic acid, alanine, aspartic acid, b-alanine, citric acid,	other C metabolisms	Phosphopantetheine-adenylyltransferase (PPAT) (EC 2.7.7.3)	AT2G18250	**x**

		fumaric acid, GABA, malic acid, maltose-turanose, myo-inositol, maltitol, quinic acid, ribose, shikimic acid, valine				
Traes_7AS_E477E850F	*down*	aconitic acid, alanine, aspartic acid, beta-alanine, fumaric acid, GABA, malic acid, maltose-turanose, myo-inositol, maltitol, quinic acid, valine	Stress	Protein DETOXIFICATION	AT4G25640	**x**
Traes_1AL_4BF6C25E1	*up*	aconitic acid, citric acid, GABA, maltose-turanose, myo-inositol, quinic acid, ribose, shikimic acid, valine	Others	Probable transmembrane GTPase FZO-like	AT1G03160	**x**
Traes_1AL_1A98A80B4	*up*	aconitic acid, GABA myo-inositol, quinic acid, ribose, shikimic acid, valine	C metabolism (photosynthesis)	Chlorophyll synthase, chloroplastic (EC 2.5.1.62)	AT3G51820	**x**
Traes_1BL_0FC423766	*up*	GABA, myo-inositol, maltitol, quinic acid, valine	Others	60S ribosomal protein L36	AT5G02440	**x**
Traes_1BL_714F4E4AC	*up*	aconitic acid, GABA, maltose-turanose, myo-inositol, quinic acid, ribose, shikimic acid, valine	Stress	Carotene epsilon-monooxygenase, chloroplastic (EC 1.14.99.45)	AT3G53130	**x**
Traes_1BL_8AC8D124C	*up*	aconitic acid, citric acid, GABA, myo-inositol, quinic acid, ribose, shikimic acid, valine	Others	50S ribosomal protein L1	AT3G63490	**x**
Traes_2AL_E7D1C8AA5	*up*	GABA, maltose-turanose, myo-inositol, maltitol, quinic acid, shikimic acid, valine	transporter	Probable inositol transporter 2	AT1G30220	**x**

Traes_2AL_FA3E7C60B	*up*	aconitic acid, GABA, isocitric acid, maltose-turanose, myo-inositol, maltitol, quinic acid, valine	Stress	Violaxanthin de-epoxidase, chloroplastic (VDE 1) (EC 1.23.5.1)	AT1G08550	**x**
Traes_2AL_20BC426B9	*up*	aconitic acid, aspartic acid, citric acid, GABA myo-inositol, maltitol quinic acid, ribose, valine	Stress	Peroxidase (EC 1.11.1.7)	AT5G17820	**x**
Traes_2AL_729CB1040	*up*	GABA, myo-inositol quinic acid, ribose, valine	C metabolism (glycolysis)	Aldose 1-epimerase (EC 5.1.3.3)	AT3G17940	**x**
Traes_2AL_E06A248EE	*up*	alanine, beta-alanine, fumaric acid, GABA myo-inositol, maltitol, quinic acid, ribose, shikimic acid, valine	Stress	Glutathione peroxidase (EC 1.11.1.9)	AT2G48150	**x**
Traes_2BL_363CA4ED8	*up*	aconitic acid, alanine, GABA, maltose-turanose, myo-inositol, maltitol, quinic acid, ribose, shikimic acid, valine	C metabolism (photosynthes is)	Ferredoxin-3	AT2G27510	**x**
Traes_2BS_FF8887BE5	*up*	aconitic acid, citric acid, GABA, maltose-turanose, myo-inositol, quinic acid, ribose, shikimic acid, valine	Others	Asparagine--tRNAligase, chloroplastic/mitochondrial	AT4G17300	**x**
Traes_2BS_7135D68C5	*up*	alanine, GABA, myo-inositol, maltitol, quinic acid, ribose	Others	Ribosomal protein L19	AXX17-AT4G02830	**x**
Traes_2BS_E5A5144E0	*up*	alanine, fumaric acid GABA, maltitol, quinic acid, ribose	Others	tRNA (guanine(26)-N(2))-dimethyltransferase		**x**
Traes_3AS_3CB8A9C01	*up*	aconitic acid, GABA, myo-inositol, quinic acid ribose, valine	Amino acid metabolism	Glutamate decarboxylase (EC 4.1.1.15)	AT1G65960	**x**
Traes_3B_00CF2A894	*up*	GABA, myo-inositol,	Stress	ATP-dependent Clp protease	AT1G66670	**x**

		quinic acid, ribose, shikimic acid, valine		proteolytic subunit 3 (EC 3.4.21.92)			
Traes_4AL_497775E8F	*up*	alanine, beta-alanine GABA, myo-inositol, Maltitol, quinic acid, ribose, shikimic acid, valine	Stress	Ferritin (EC 1.16.3.1)	AT3G56090		**x**
Traes_4BS_8D508569E	*up*	aconitic acid, GABA, myo-inositol, quinic acid, ribose, shikimic acid, valine	Others	Pentatricopeptide repeat-containing protein At3g06430	AT3G06430		**x**
Traes_5AL_955D2899A	*up*	aconitic acid, alanine GABA, maltose-turanose, myo-inositol maltitol, quinic acid, ribose, shikimic acid, valine	Stress	Pectin acetylesterase (EC 3.1.1-)	AT5G23870		**x**
Traes_5AS_6D0CC3B1F	*up*	GABA, maltose-turanose, myo-inositol, Maltitol, quinic acid, shikimic acid, valine	Others	60S ribosomal protein L36	AT5G02440		**x**
Traes_5BL_304FAFA26	*up*	GABA, myo-inositol, maltitol, quinic acid, ribose, valine	Stress	Lipoxygenase (EC 1.13.11-)	AT3G45140		**x**
Traes_6AL_D13D9D951	*up*	aconitic acid, GABA, maltose-turanose, myo-inositol, maltitol, quinic acid, ribose, valine	stress	Serine carboxypeptidase-like 34(EC 3.4.16-)	AT5G23210		**x**
Traes_6AL_58B6B7319	*up*	aconitic acid, alanine, aspartic acid, citric acid, GABA, malic acid, maltose-turanose, myo-inositol, maltitol, quinic acid, ribose, valine	transporter	Bidirectional sugar transporter SWEET	AT3G16690		**x**
Traes_6AS_0F0809F96	*up*	aconitic acid, GABA, maltose-turanose, myo-inositol, maltitol,	stress	Probable inactive linolenate hydroperoxide lyase	AT4G15440		**x**

		quinic acid, ribose, shikimic acid, valine				
Traes_6AS_87906149C	*up*	aconitic acid, GABA, maltose-turanose, myo-inositol, quinic acid, ribose, shikimic acid, valine	others	Protein Chlororespiratory Reduction 6 (CRR6)	AT2G47910	**x**
Traes_7AL_356F6FF0E	*up*	aconitic acid, citric acid, GABA, maltose-turanose, myo-inositol, maltitol, valine	others	Blue-light photoreceptor PHR2	AT2G47590	**x**
Traes_4AL_320703FD2	*up*	aconitic acid, alanine, aspartic acid, fumaric acid, GABA, malic acid, myo-inositol, maltitol, quinic acid, ribose, valine	other C metabolisms	Xyloglucan endotransglucosylase/hydrolase (EC 2.4.1.207)	AT2G06850	**x**
Traes_7AL_1B1FBCDE4		aconitic acid, alanine, beta-alanine, citric acid, GABA, malic acid maltose-turanose, myo-inositol, maltitol, quinic acid, ribose, valine				
Traes_7AL_8C4A9BEBF		aconitic acid, alanine, aspartic acid, b-alanine, citric acid, fumaric acid, GABA, malic acid, maltose-turanose, myo-inositol, maltitol, quinic acid, ribose, shikimic acid, valine				
Traes_7AS_020CCE3DB	*up*	aconitic acid, citric acid, GABA, maltose-turanose, myo-inositol, maltitol,	Stress	Peroxiredoxin Q, chloroplastic (EC 1.11.1.15)	AT3G26060	**x**

		quinic acid, ribose, valine					
Traes_7AS_545BDB6F1	*up*	aconitic acid, GABA, maltose-turanose, myo-inositol, quinic acid, ribose, shikimic acid, valine	Others	Alanine--tRNA ligase, chloroplastic/mitochondrial (EC 6.1.1.7)	AT5G22800		**x**
Traes_7BL_A42D6C984	*up*	aconitic acid, GABA, maltose-turanose, myo-inositol, maltitol, quinic acid, ribose, valine	Stress	Superoxidedismutase [Cu-Zn] 2 (EC 1.15.1.1) SOD2	AT2G28190		**x**
Traes_7BL_B5491A48C	*up*	aconitic acid, alanine, citric acid, GABA, malic acid, maltose-turanose, myo-inositol, maltitol, quinic acid, ribose, shikimic acid, valine	Amino acid metabolism	D-3-phosphoglycerate dehydrogenase (EC 1.1.1.95)	AXX17-AT4G39120		**x**
Traes_7BL_C35CD97E7	*up*	aconitic acid, alanine, citric acid, GABA, maltose-turanose, myo-inositol, maltitol, quinic acid, ribose, shikimic acid, valine	C metabolism (photosynthesis)	Delta-aminolevulinic acid dehydratase (EC 4.2.1.24)	AXX17-AT1G63860		**x**
