# Supplementary material for: Comparative Analysis Based on Transcriptomics and Metabolomics Data Reveal Differences between Emmer and Durum Wheat in Response to Nitrogen Starvation
Source: Int J Mol Sci. 2021 Apr 30;22(9):4790. doi: 10.3390/ijms22094790 (PMC8124848; doi:10.3390/ijms22094790)
Supplement: Supplementary file 1 [file ijms-22-04790-s001.zip › Suppl.Table S9.pdf]

Table S9. DEGs of networks affected by environment.

	DEGs	(-N)	(+N)
**Common central role in both network**	Traes_2BL_CCD296233	up	up
**Common central role in durum wheat network**	Traes_5BL_BD895CF84	down	
**Common central role in emmer network**	Traes_2AS_F485758C1	down	down
	Traes_6AS_FD8F6B539	down	
**Specific durum wheat network**	Traes_1BL_9772C6F45	down	
	Traes_2BS_7135D68C5	up	
	Traes_2AL_20BC426B9		down
	Traes_5AL_07C125B1C		up
	Traes_5BL_FF9AA4B58		up
	Traes_6AS_0C1D497EA		up
	Traes_7AL_1B1FBCDE4	down	down
**Specific emmer network**	Traes_1AL_5F0F4FAFB	up	
	Traes_1AL_8E8E7401B	down	
	Traes_2AS_34272605C	down	
	Traes_4AS_4C25DF6F4	down	
	Traes_7AS_9A01E6B17	up	
	Traes_7AS_A80BE362A	down	
