# Supplementary figures and images for: Comparative Analysis Based on Transcriptomics and Metabolomics Data Reveal Differences between Emmer and Durum Wheat in Response to Nitrogen Starvation
Source: Int J Mol Sci. 2021 Apr 30;22(9):4790. doi: 10.3390/ijms22094790 (PMC8124848; doi:10.3390/ijms22094790)

Figure S2.

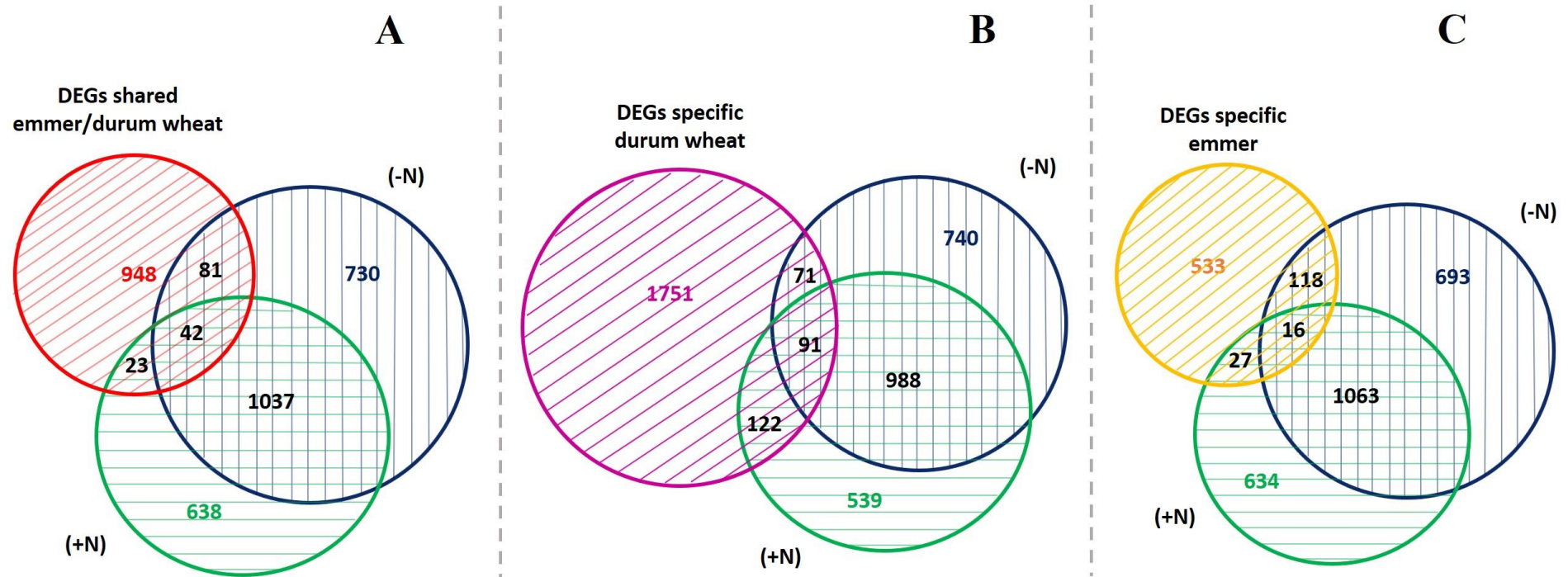

Supplement: Supplementary file 1 [file ijms-22-04790-s001.zip › Suppl. Figure S2.pdf]
